# Rapid Path Planning Algorithm for Percutaneous Rigid Needle Biopsy Based on Optical Illumination Principles

**DOI:** 10.3390/s25072137

**Published:** 2025-03-28

**Authors:** Jian Liu, Shuai Kang, Juan Ren, Dongxia Zhang, Bing Niu, Kai Xu

**Affiliations:** 1 School of Mechanical Engineering, Shanghai Jiao Tong University, Shanghai 201100, China; maoforest@163.com; 2 Shanghai Simple Touch Technology Co., Ltd., Shanghai 201600, China; kangshuai@simple-touch.cn (S.K.); renjuan@simple-touch.cn (J.R.); zhangdx@simple-touch.cn (D.Z.)

**Keywords:** biopsy path planning, optical acceleration, machine learning, segmentation, graphics processing unit (GPU) optimization, clinical evaluation

## Abstract

Optimal needle trajectory selection is critical in biopsy procedures to minimize tissue damage and ensure diagnostic accuracy. Timely trajectory planning is essential, as it relies on preoperative CT imaging. Prolonged processing times increase the risk of patient movement, rendering the planned path invalid. Traditional methods relying on clinician expertise or slow algorithms struggle with complex anatomical modeling for structures such as blood vessels. We introduce a novel method that reframes trajectory planning as an optimal puncture site identification problem by leveraging optical principles and computer rendering. A 3D model of key anatomical structures is reconstructed from CT images and segmented using SegResNet (average Dice similarity coefficient of 0.9122). A virtual light source positioned at the target illuminates the space, assigning distinct absorption coefficients to tissues based on needle permissibility and risk. Diffuse reflection simulates needle angle, and accumulated absorption represents depth, capturing puncture constraints. This simulation generates a grayscale map on the skin surface, highlighting candidate puncture sites. Furthermore, we employ a random forest-based method to model clinician preferences. This model analyzes an RGB image derived from the grayscale distribution to automatically select the optimal path and determine the needle entry point. The experimental evaluation demonstrates an average computation time of just 1.905 s per sample, which is significantly faster than traditional methods that require seconds to minutes. Moreover, clinical assessment by a thoracic surgeon found that 78% of the recommended paths met clinical standards, with 0% deemed unsatisfactory. These findings suggest that our method provides a rapid, intuitive, and reliable decision-support tool, improving biopsy safety and efficiency.

## 1. Introduction

Computed tomography (CT)-guided percutaneous puncture, a cornerstone of modern minimally invasive medicine, has become indispensable for diagnosis and treatment across a wide range of conditions, particularly within chest and abdominal organs like the lung and liver [1,2]. Cancers in these regions represent a staggering global health crisis, demanding diagnostic and therapeutic solutions that are both minimally invasive and maximally effective [3,4]. Given these pressing health challenges, ensuring that diagnostic and therapeutic procedures are both accurate and minimally invasive becomes paramount.

Percutaneous tumor biopsies, which commonly employ thin, rigid needles to extract small tissue samples for microscopic examination, are crucial for early and definitive cancer diagnosis and facilitate personalized treatment strategies [5,6]. Similarly, percutaneous ablation techniques utilize rigid needles to deliver energy (e.g., radiofrequency or microwave) to destroy tumor tissue in situ [7,8]. Rigid needles are favored in these interventions for their proven efficacy, ease of use, and cost-effectiveness compared to more complex flexible needles, even though their limited maneuverability poses challenges [9,10,11,12].

A fundamental difficulty arises from the fact that internal organs such as the lung and liver are deeply situated and cannot be directly visualized. Consequently, conventional manual puncture procedures rely heavily on clinician experience and are essentially performed without direct visualization, which introduces significant risk and variability [1,2,13,14]. In this context, the clinical effectiveness of both biopsies and ablations is tightly coupled with the precision of the puncture path. Even minor inaccuracies in needle placement can compromise diagnostic yield, therapeutic accuracy, and patient safety, potentially leading to severe complications like pneumothorax, hemorrhage, and nerve injury [5,15,16,17].

To address these limitations, CT-guided percutaneous puncture navigation robots have emerged as an innovative integration of advanced medical imaging, sophisticated path planning algorithms, and robotic-assisted interventions. These systems are designed to markedly improve procedural safety and precision. Their workflow generally comprises three principal stages:

(1) **Image Acquisition and Target Identification:** High-resolution CT images are acquired and imported into the system, where either the operator or dedicated software delineates the puncture target and maps the surrounding anatomical structures. This step includes assessing tissue traversability to ensure that the planned trajectory avoids regions that could trigger adverse or even fatal complications.
(2) **Trajectory Planning:** Given the rigidity of the needle, the system identifies one or more safe entry points in three-dimensional space relative to the target. It then computes a straight-line trajectory that avoids critical structures (e.g., bones, blood vessels, airways, and fissures), thereby ensuring a safe path for needle insertion.
(3) **Physical Trajectory Establishment and Execution:** The virtual path is transferred to the physical environment, where the needle is advanced along the pre-planned trajectory either manually by the operator or automatically via robotic control, ensuring precise needle placement.

The emergence of robotic percutaneous interventions holds immense promise for enhancing the precision and consistency of percutaneous biopsy and ablation procedures [1,2]. However, two critical challenges remain. First, both conventional manual puncture procedures and robotic systems that lack automated planning capabilities still rely on laborious, time-intensive manual analysis of CT images by specialized physicians [18,19]. Although clinician expertise is indispensable, such manual approaches are inherently subjective and prone to inter-operator variability, often resulting in suboptimal trajectories and necessitating multiple puncture attempts, thus increasing patient discomfort and procedure duration [8].

Second, while robotic systems that incorporate automated path planning aim to enhance precision and consistency, they frequently encounter issues related to high computational loads and extended processing times. In time-sensitive CT-guided interventions—where patient immobility is crucial—delays in computation can lead to discrepancies between pre-calculated and actual needle trajectories. This is particularly problematic when even minor delays can result in patient movement or tissue shifts (e.g., due to respiratory motion), ultimately increasing the risk of medical errors [2,20].

Addressing these challenges is essential for advancing CT-guided percutaneous interventions and is critical for achieving rapid, robust, and adaptable needle path planning that is reliably applicable in real-world scenarios.

The convergence of advanced computing, image processing, and artificial intelligence (AI) offers promising avenues to overcome these limitations. Researchers are actively developing computer-aided surgical planning systems that can efficiently process complex imaging data in near-real time, automatically extract anatomical information, and optimize paths under multiple clinical constraints [21,22,23]. These systems aim to balance objectives such as minimizing path length while maximizing safety margins, thereby alleviating clinician workload and enhancing both accuracy and objectivity in robotic percutaneous interventions [2,6,8,24,25].

However, persistent challenges remain. Many state-of-the-art algorithms demand significant computational resources and require several minutes to compute a puncture path for a single patient case [16,26]. For patients and clinicians, such delays are unacceptable and potentially hazardous; prolonged waiting times increase the risk of patient movement or tissue shifts, leading to discrepancies between preoperative CT images and the actual surgical scenario, which can precipitate medical errors [27,28,29]. Furthermore, accurately and efficiently modeling intricate vascular structures imposes additional computational burdens [9,30,31]. Traditional approaches that require precise delineation of the course and diameter of each vessel are computationally intensive [21,32], whereas overly conservative methods that designate vessels as forbidden zones can unnecessarily restrict viable trajectories [21,25]. In practice, many studies simplify vascular modeling by treating blood vessels as impassable regions, imposing a hard constraint [21,25,33,34]. Although this simplification streamlines the modeling process, it also eliminates numerous potential paths—since, under certain conditions, small vessels can be safely traversed [35,36,37]. This dilemma highlights the core challenge: how to achieve accurate vascular safety assessment without incurring excessive computational cost. Consequently, many sophisticated multi-objective optimization algorithms and path recommendation systems struggle to provide rapid solutions [5,15,17,21,25], impeding real-time responsiveness in dynamic surgical environments.

To address these critical challenges, this study proposes and validates a precise and rapid rigid needle path planning method for robot-assisted percutaneous puncture. In recent years, the rapid advancement of graphics processing unit (GPU) technology and computational lighting simulation techniques—such as the NVIDIA^®^ OptiX (NVIDIA Corporation, Santa Clara, CA, USA) engine, which leverages CUDA^®^ (NVIDIA Corporation, Santa Clara, CA, USA) for efficient ray tracing—has dramatically accelerated computational processes [38]. Leveraging the principle that light travels in straight lines, analogous to rigid needle trajectories, we hypothesized that optical principles could be effectively applied to needle path planning for enhanced computational efficiency and precision. The omnidirectional propagation of light in 3D space further mirrors the clinical scenario of fixing a target and searching for an optimal entry point in three dimensions. Inspired by this analogy, our approach frames lung puncture path planning as an optical illumination computation task. This study introduces two key improvements to address the challenges of rigid needle path planning in robot-assisted percutaneous puncture:

(1) **Ultra-fast Path Computation:** Leveraging optical principles and GPU acceleration, our method achieves ultra-fast path calculations by simulating light absorption and reflection, offering a computationally lightweight solution that circumvents complex geometric computations.
(2) **Simplified Vascular Modeling:** Our approach simplifies vascular modeling by assigning light absorption parameters to vascular voxels, accumulating vascular thickness along the path without explicit vessel segmentation, thus balancing computational efficiency and vascular safety.

Although demonstrated using organ segmentation models as a proof of concept, our approach is compatible with any viable segmentation algorithm. While our focus is on lung puncture, the method is readily adaptable to other organs (e.g., liver, kidneys) and procedures (biopsies, ablations). This innovation promises to enable safer, faster, and more precise percutaneous interventions by addressing critical issues related to procedure time, patient immobility, and the need for real-time path adaptation in dynamic robotic environments.

## 2. Materials and Methods

This section details the implementation of our optical illumination-based needle path planning algorithm, structured in four core components. First, we analyzed rigid needle percutaneous puncture constraints, categorizing them as hard and soft to theoretically ground the algorithm design. Second, experimental data preparation was crucial for development and testing. We built segmentation models for organs, tissues, and lesions, providing 3D spatial data for path planning. Subsequently, we detailed the optical model principles of the algorithm, including its construction, constraint representations, and the light intensity model. Finally, we presented the implementation specifics: data preprocessing, intensity calculation, path optimization, and validation.

### 2.1. Constraints of Rigid Needle

In the context of percutaneous lung biopsy using a rigid needle, the approach to path planning is fundamentally based on constraint-based restrictions to ensure patient safety and procedural effectiveness. It is essential to distinguish between hard constraints, which are absolute and non-negotiable, and soft constraints, which are desirable but can be relaxed if necessary. This distinction allows for a balance between safety and flexibility in the planning process.

#### 2.1.1. Hard Constraints

**Figure 1:**
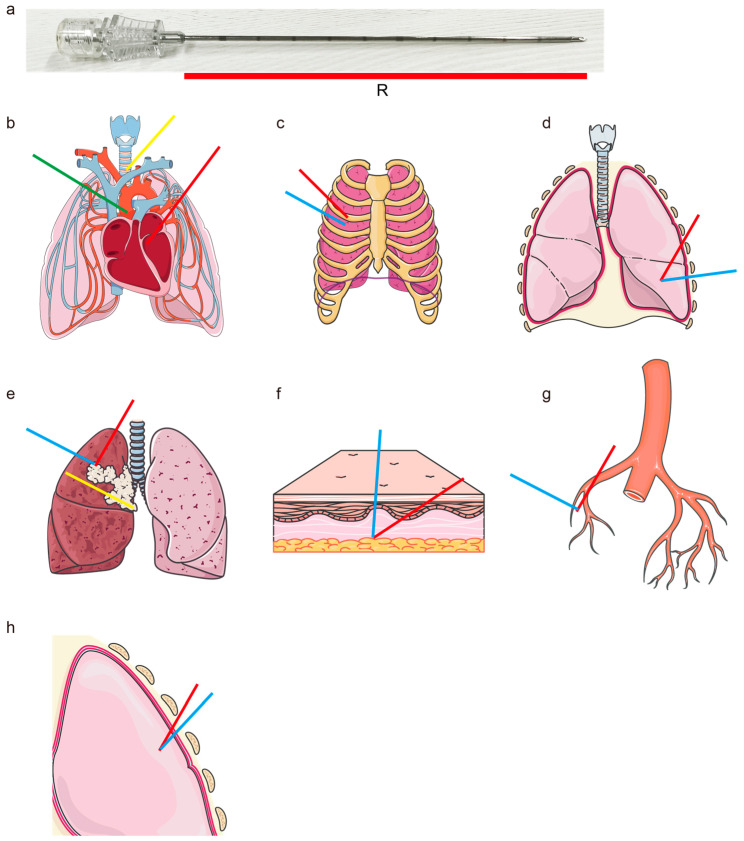
Illustrative examples of rigid needle path planning considerations in percutaneous interventions. (**a**) Needle Rigidity Assumption: A photograph of a biopsy needle. In clinical practice, any deformation of the needle during puncture is typically disregarded, assuming a rigid, linear trajectory within the tissue. The red line indicates the effective working length (R) of the needle. (**b**) Critical Organ and Tissue Avoidance: Trajectories are shown with various colors; the red path traverses the heart, the green path violates major blood vessels, and the yellow path penetrates the trachea. Each of these trajectories is contraindicated. (**c**) Bone Avoidance: The red path punctures the rib inappropriately, whereas the blue path successfully avoids bony structures. (**d**) Lobe-Specific Targeting: The red path, intended for the left lower lobe, undesirably crosses into the left upper lobe, causing unintended damage and increasing the risk of pneumothorax. In contrast, the blue path remains confined to the left lower lobe, representing a preferable trajectory. (**e**) Path Length Constraints: An irregular white shape denotes a tumor. The yellow path exceeds the effective length R of the needle, rendering it physically infeasible since it would necessitate the tail of the needle remaining inside the body. Moreover, the red path, being significantly longer than the blue path, suggests greater tissue trauma, and is suboptimal compared to the shorter blue path. (**f**) Skin Entry Angle: The red path features an excessively oblique skin entry angle, while the near-perpendicular blue path represents a more favorable approach. (**g**) Major Vessel Avoidance: The blue path selectively traverses only minor vessels, making it acceptable. Conversely, the red path intersects multiple major blood vessels; thus, it is contraindicated. (**h**) Distance from Critical Structures: A schematic illustrates that the red path offers insufficient distance from the rib, posing a higher risk in clinical practice, whereas the blue path maintains a safer margin.

- **Straight Path for Rigid Needle:** The path must be straight to accommodate the rigidity of the needle (Figure 1a).
- **Avoidance of Vital Organs/Tissues:** The path must avoid the heart and major blood vessels to prevent severe complications (Figure 1b).
- **Avoidance of Bones:** The needle must not penetrate the ribs or other bones to prevent fractures or physical obstruction (Figure 1c).
- **Only Pass Through Target Lung Lobe:** The path must stay within the target lung lobe and avoid crossing into non-target lung lobes, ensuring no traversal of interlobar fissures (Figure 1d).
- **Path Length Must Be Less Than Needle Length:** The path length must be within the physical limits of the needle to ensure it can reach the target site effectively (Figure 1e).

#### 2.1.2. Soft Constraints

- **Minimize Path Length:** Shorter paths are preferred to reduce the risk of complications (Figure 1e).
- **Maximize Angle with Skin:** The angle of insertion with the skin should be as large as possible for easier needle insertion (Figure 1f).
- **Prefer Smaller and Fewer Vessels:** If it is necessary to pass through vessels, the path should prefer smaller and fewer vessels to minimize the risk of bleeding or other complications (Figure 1g).
- **Maximize Distance from Vital Organs/Tissues:** The path should be optimized to maintain the maximum feasible distance from vital organs and tissues, thereby minimizing the risk of unintended damage (Figure 1h).

### 2.2. Segmentation of Organs, Tissues, and Lesions

#### 2.2.1. Datasets

The dataset utilized in this study was sourced from the publicly available LUNA16 dataset, which comprises a comprehensive collection of lung CT scans. Specifically, the dataset includes 888 CT scans and 1186 annotations from 888 distinct patients, each annotated with detailed information regarding lung nodules, which are crucial for lung cancer screening. The dataset was preprocessed to ensure consistency and compatibility with the segmentation model, involving standard steps such as normalization and resampling to the target resolution of isotropic voxels.

#### 2.2.2. Data Annotation and Training

The annotation process was outsourced to a team of experienced radiologists who meticulously delineated the regions of interest, including organs, tissues, and lesions, using a state-of-the-art annotation tool. The annotations were rigorously reviewed to ensure high accuracy and consistency. We have annotated a total of 250 cases of data for this study, covering organs or tissues related to percutaneous lung puncture, such as lung lobes, pulmonary blood vessels, the heart, the liver, the spleen, the lungs, they kidneys, the pancreas, the body, and the airway tree. The labels of the pulmonary nodules used the original labels in the LUNA16 dataset [39].

To ensure safe path planning, we adopted a coarse–fine segmentation strategy for precise organ segmentation. First, we trained a lightweight, low-resolution ROI (Region of Interest) extractor to distinguish between regions inside and outside the body, effectively reducing external noise. Next, a high-resolution, complex model was applied to the extracted regions to segment organs and tissues in detail. We further improved the segmentation by fine-tuning the patch size according to the anatomical characteristics of the target organs. Finally, the outputs of both models were combined through an ensemble approach.

We used SegResNet as our baseline for segmentation. This U-Net-like architecture integrates residual (Res) blocks in both the encoder and decoder paths, enhancing feature extraction and stabilizing training. To assess its key components, we conducted ablation and comparative studies:

**Residual Module Ablation:** We replaced the residual blocks with basic convolutional blocks—keeping all else constant—thus converting SegResNet into a standard 3D-UNet and quantifying the residual modules’ impact.

**Transformer Encoder Experiment:** We substituted the convolutional encoder with a Transformer encoder employing self-attention while retaining the decoder. This produced a UNETR-like model, allowing us to evaluate the benefits of Transformer integration.

All experiments were performed using the nnU-Net [40] framework with the 3d_lowres (3 mm isotropic voxels) and 3d_fullres (1 mm isotropic voxels) configurations. The models were trained on 200 annotated images, with a batch size of 2 over 1000 epochs, optimized using Adam (initial LR 0.01) and Dice loss.

#### 2.2.3. Validation of Segmentation Models

The trained segmentation model was validated using a hold-out validation set comprising 50 images not included in the training phase. The validation process involved computing the standard segmentation metrics to assess the performance of the model.

Dice Similarity Coefficient (DSC): This quantifies 3D volumetric overlap between the segmentation and ground truth.

(1) $$DSC=\frac{2\left|A\cap B\right|}{\left|A\right|+\left|B\right|},$$

where A and B represent the 3D volumes of the segmentation results and the ground truth region. Here, $\left|A\right|$ and $\left|B\right|$ denote the number of voxels in the corresponding volumes.

Hausdorff Distance (HD): This measures the maximum boundary distance between the segmentation and the ground truth. We use the 95th percentile (HD95) to reduce outlier effects.

(2) $$HD(A,B)=max\left(\underset{a\in A}{max}\underset{b\in B}{min}∥a-b∥,\underset{b\in B}{max}\underset{a\in A}{min}∥b-a∥\right),$$

where A represents the set of surface points of the segmentation result, and B represents the set of surface points of the ground truth region. a∈A indicates that point a is an element of set A, and b∈B indicates that point b is an element of set B. ∥ ∥ denotes the distance between two points.

Average Surface Distance (ASD): This represents the mean shortest distance between the two surfaces, reflecting segmentation shape accuracy.

(3) $$ASD=\frac{1}{\left|S_{1}\right|}\underset{s_{1}\in \;S_{1}}{\sum }\underset{s_{2}\in \;S_{2}}{min}∥s_{1}-s_{2}∥+\frac{1}{\left|S_{2}\right|}\underset{s_{2}\in \;S_{2}}{\sum }\underset{s_{1}\in \;S_{1}}{min}∥s_{2}-s_{1}∥,$$

where $S_{1}$ and $S_{2}$ represent the surfaces of the segmentation results and the ground truth, respectively, and $s_{1}\in \;S_{1}$ and $s_{2}\in \;S_{2}$.

True positives (TPs) refer to target voxels correctly identified by the segmentation model, while False Positives (FPs) are background voxels mistakenly classified as the target. True negatives (TNs) denote background voxels accurately recognized as non-target, and False Negatives (FNs) are target voxels that the model fails to detect, being incorrectly labeled as background.

Specificity (Spec): This indicates the proportion of true negative voxels correctly identified.

(4) $$\mathrm{Spec}=\frac{TN}{TN+FP}.$$

Sensitivity (Sens): This measures the proportion of true positive voxels correctly segmented.

(5) $$\mathrm{Sens}=\frac{TP}{TP+FN}.$$

Relative Volume Error (RVE): This expresses the percentage difference between the segmented and true volumes (positive for over-segmentation, negative for under-segmentation).

(6) $$\mathrm{RVE}=\frac{V_{\mathrm{seg}}-V_{\mathrm{gt}}}{V_{\mathrm{gt}}}\times 100\%,$$

where $V_{\mathrm{seg}}$ represents the volume of the segmented region produced by the model, whereas $V_{\mathrm{gt}}$ denotes the volume of the true region as defined by the ground truth annotations.

#### 2.2.4. Robustness Test of Segmentation Models

The data collection and annotation processes for the Tianchi Lung Nodule dataset [41], LNDb dataset [42], and our internal clinical dataset were consistent with those described for the LUNA16 dataset. To evaluate the robustness and generalization of our segmentation model, we annotated these datasets following the same protocols. Importantly, these datasets were solely used for testing purposes and were not included in the training phase. Segmentation metrics, including average sensitivity, average specificity, average DSC, average RVE, average HD95, and average ASD, were computed to assess model performance across different data distributions.

### 2.3. Principles of an Optical Illumination-Based Path Planning Algorithm

#### 2.3.1. Basic Concept

To efficiently determine a starting point for rigid needle path planning to a fixed target, we employ an optical model, drawing an analogy to 3D illumination from a point light source. Given the straight path constraint of rigid needles and considering all 3D points as potential starting positions, we simulate light propagation from the target. In this model, the human body is conceptualized as a 3D ‘light box’, with tissues assigned varying light absorption coefficients based on puncture risk: opaque for high-risk tissues (bone, non-target organs), transparent for safe tissues (muscle, target lesions), and partially translucent for vessels. By simulating reverse light emission from the target and projecting cumulative absorption onto a spherical shell, we generate a brightness distribution. Brighter regions indicate favorable, constraint-compliant entry points, simplifying optimal path identification. Crucially, this optical modeling approach allows us to leverage existing illumination algorithms and hardware acceleration techniques, significantly enhancing computational speed for practical application.

#### 2.3.2. Biopsy Needle Length Constraint

**Figure 2:**
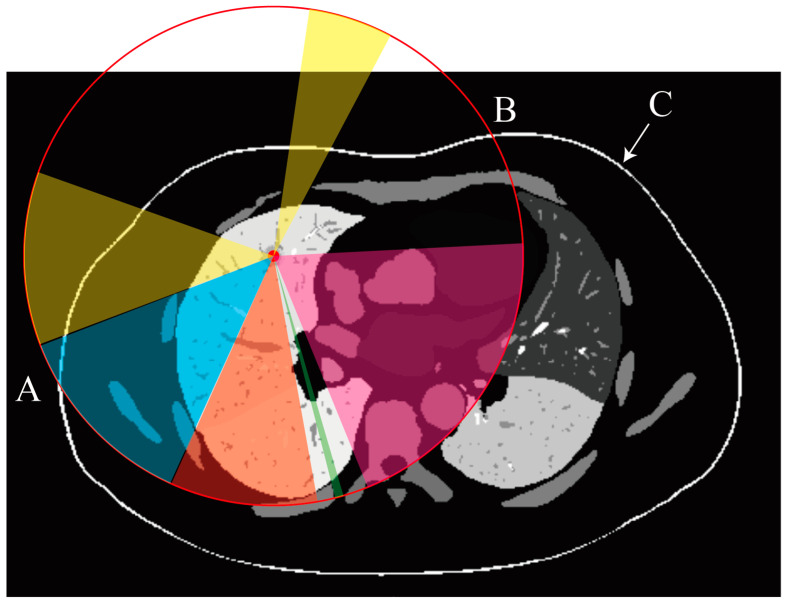
Two-dimensional cross-sectional view showing the potential puncture paths in a biopsy procedure.

Figure 2 presents a 2D cross-sectional view of a region containing potential puncture paths. Grayscale values indicate the segmentation labels for different organs or tissues. The red point marks the puncture target, and the red circle represents a sphere with a radius corresponding to the length of the biopsy needle (R). Feasible puncture paths are constrained to lie within the region between points A and B, ensuring that the needle can reach the target. The colors represent various constraints: yellow indicates feasible areas, blue indicates areas obstructed by the rib, orange denotes regions where the needle would cross multiple lung lobes, green signifies insufficient needle length, and pink highlights areas blocked by the heart or major arteries. Point C and the white arrow indicate the skin mask, while points A and B mark the intersections of the red circle with the skin surface.

We first address the biopsy needle length constraint using a method that efficiently reduces data volume and enhances computational efficiency.

To describe the candidate region set C, we consider the following mathematical formulation:

Given a target point, $T=\left(x_{t},y_{t},z_{t}\right)$ and a sphere of radius R centered at T, defined by the following equation:

(7) $${\left(x-x_{t}\right)}^{2}+{\left(y-y_{t}\right)}^{2}+{\left(z-z_{t}\right)}^{2}\leq R^{2},$$

where $S\left(x,y,z\right)$ represents the skin surface, with $S\left(x,y,z\right)>0$ indicating points outside the skin. The candidate region C consists of all points within the sphere where the tail of the needle can be placed, satisfying the following conditions:

1. The tail is outside the skin: $S\left(x,y,z\right)>0$.
2. The line segment L from the tail to T intersects the skin surface $S\left(x,y,z\right)=0$.
3. The tail position is within the visible skin region of the CT scan.

Formally, the candidate region C is defined as

(8) $$C=\left\{\left(x,y,z\right)|\begin{array}{c} (x-x_{t}{)}^{2}+(y-y_{t}{)}^{2}+(z-z_{t}{)}^{2}\leq R^{2},\\ S(x,y,z)>0,\\ L\cap \{S(x,y,z)=0\}\neq \emptyset ,\\ (x,y,z)\;is\;in\;the\;visible\;skin\;region\;of\;the\;CT\;scan \end{array}\right\}.$$

This formulation ensures that the tail of the needle is positioned outside the skin, the path to the target intersects the skin, and the position is within the scanned and visible region of the CT data.

Furthermore, regions outside the sphere are excluded because they are irrelevant given the needle length constraint. This exclusion significantly reduces the data processed by the model, ensuring that only relevant regions are considered. This approach is particularly effective when CT images do not include the skin due to scanning limitations.

#### 2.3.3. Organ and Vessel Avoidance and Depth Regulation

In this section, we address the critical constraints of avoiding important organs, navigating through vasculature, and regulating puncture depth to ensure safe and effective needle insertion. We introduce a comprehensive risk assessment framework that accounts for the complexities of vessel traversal and tissue thickness.

**a.**  **Organ Avoidance**

To ensure the safety of critical anatomical structures during needle insertion, we identify and mark organs such as the ribs and trachea as opaque within our model. This opacity designation serves to exclude any needle paths that intersect these high-risk areas. The rationale behind this approach is to minimize the potential for damage to these vital organs, which could lead to complications. The determination of opacity is based on the anatomical importance and sensitivity of each organ, ensuring that only safe paths are considered.

**b.**  **Vessel Risk Assessment**

Navigating through the pulmonary vessels presents a unique challenge due to the densely packed vasculature in certain regions. Traditional methods for assessing vessel characteristics, such as diameter, grading, direction, and density, are often cumbersome and computationally intensive, making them less practical for real-time applications. In contrast, our light absorption model provides a streamlined and efficient solution for assessing the risk associated with vessel traversal. By utilizing this model, we can simplify the risk assessment process, reducing the complexity and potential errors associated with traditional methods.

**c.**  **Puncture Depth Regulation**

Regulating the depth of needle insertion is crucial for achieving the desired clinical outcome while minimizing risks. We define an acceptable range for needle insertion depth based on the properties of the target tissue. To simulate and control this depth, we employ cumulative light absorption data, which correlate with tissue thickness. By integrating these data into our path evaluation model, we can accurately estimate the insertion depth and penalize or exclude paths that fall outside the specified range. This approach enhances the precision of depth regulation, thereby minimizing the risk of over- or under-penetration.

**d.**  **Integration into Path Evaluation**

The integration of organ and vessel avoidance, along with puncture depth regulation, is achieved through a cohesive framework that modulates light intensity on the shell by adjusting the transparency of different organs or tissues. Regions that should be avoided or traversed minimally are assigned penalty scores, which are determined by their transparency or path length. This ensures that these regions contribute less to the final light intensity score, guiding the algorithm to select safer paths.

We assign influence coefficients to tissues or organs along the needle insertion path, with tissues of equal importance receiving the same coefficient. These coefficients are then assigned to the voxels in the CT image, transforming it into a three-dimensional matrix of influence coefficients. The Beer–Lambert law is applied to model the effect of tissue length on path evaluation, relating the absorbance $A_{i}$ of a tissue to the product of its absorption coefficient $k_{i}$ and the length $l_{i}$ of the tissue through which the light (or needle) passes:

(9) $$A_{i}=-\log_{10}\left(\frac{I_{t}}{I_{0}}\right)=k_{i}\cdot l_{i}\cdot c_{i}.$$

In our model, we simplify this by omitting the concentration term $c_{i}$, as it is not relevant to our application. Consequently, we derive the following two formulas:

(10) $$A_{i}=-\log_{10}\left(\frac{I_{t}}{I_{0}}\right)=k_{i}\cdot l_{i}$$

and

(11) $$I_{t}=I_{0}\cdot 10^{-k_{i}\cdot l_{i}}.$$

**Figure 3:**
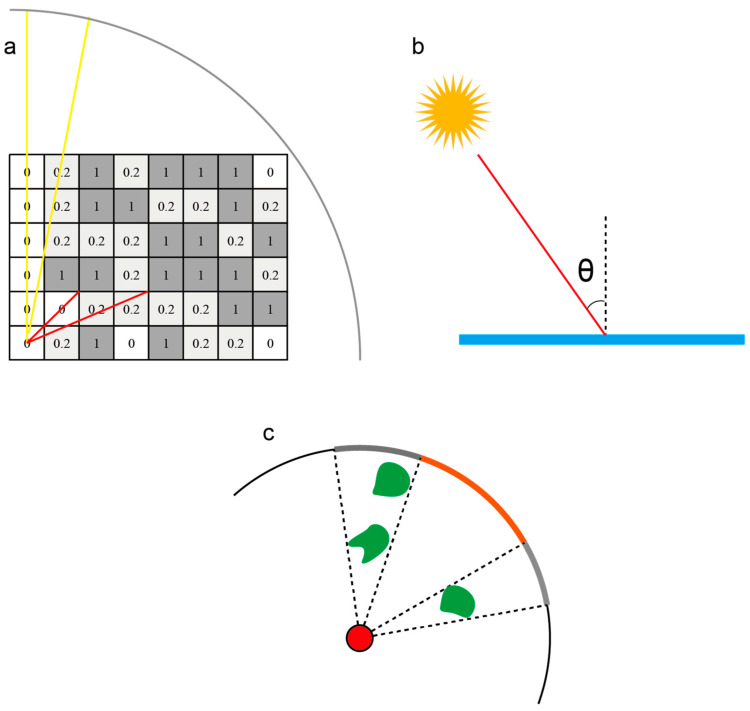
Optical illumination model for percutaneous rigid needle biopsy path planning. (**a**) Demonstration of the developed absorption model. Absorption values are assigned to organ and tissue segmentation arrays according to their risk levels. The light blocking and absorption effects are calculated, and the resulting light intensity distribution is shown on a spherical screen. (**b**) Schematic of the Lambertian lighting model used to compute the skin angle. The light, traveling along the red path, strikes the skin surface (blue), forming an angle θ with the surface normal. The diffuse reflection intensity is calculated based on the angle of incidence. (**c**) Demonstration of safe and risky needle insertion paths. The orange area on the spherical shell represents the acceptable range for potential needle insertion paths. Paths closer to the orange boundary are nearer to high-risk obstacles (green areas). Morphological dilation is applied to the obstacles to ensure that the physical distance between the path and the obstacles exceeds a threshold (e.g., 2 mm). Paths nearer to the center of the safe window are preferred to maximize the distance from obstacles.

These equations allow us to linearly quantify the effect of tissue length on the evaluation of the needle insertion path, ensuring that the integration of organ avoidance, vessel risk assessment, and puncture depth regulation is both accurate and efficient. Figure 3a illustrates the absorption model we have developed. Absorption values, assigned to segmentation arrays of various organs or tissues according to their respective risk levels, allow for the calculation of light blocking and absorption. The final light intensity distribution is then computed on a spherical screen.

#### 2.3.4. Skin Angle Constraints

To address the skin angle constraints, we employ a simplified reflection model inspired by the Lambertian reflection principle, which is typically used in computer graphics to simulate diffuse light reflection. Given the computational complexity of calculating angles for every potential entry point on the large surface area of the skin and the challenge of integrating variables with different dimensions, such as angle, length, path feasibility, and light intensity, this model offers a practical solution. By treating the skin as a diffuse reflector, we can compute a “feasibility intensity” for each potential entry point, which correlates with the angle of needle insertion. The Lambertian model posits that the intensity of reflected light is proportional to the cosine of the angle between the surface normal and the incoming light direction, expressed as

(12) $$I=I_{0}\cos\left(\theta \right),$$

where I is the resulting intensity, $I_{0}$ is the source intensity, and θ is the angle between the surface normal and the light direction. Figure 3b illustrates the Lambertian lighting model used for simulating the skin angle calculation. The light follows the red path and strikes the blue surface (representing the skin) at an angle θ. The diffuse reflection intensity is then calculated based on this angle.

#### 2.3.5. Constraints on Distance from Obstacles

In this model, obstacles—such as critical high-risk organs or tissues—are represented as opaque or nearly opaque regions, resulting in black or dark gray areas on the spherical shell. These areas correspond to the projections of the obstacles. The boundaries of these dark regions represent the projections of the obstacles, while the boundaries of the highlighted areas denote the limits of the safe regions. The highlighted areas serve as candidate regions for safe needle insertion paths.

Paths near these safe boundaries are deemed riskier due to their proximity to high-risk organs. Consequently, when selecting a needle insertion path, it is advisable to choose paths that are as far away from these boundaries as possible. As shown in Figure 3c, the acceptable range is confined within the orange-colored area on the spherical screen. The closer a path is to the edge of this orange region, the nearer it is to the green obstacles.

However, the distances measured on the spherical shell do not directly correspond to the actual physical distances from the obstacles, as these distances are influenced by the depth of the obstacles and are inversely proportional to it.

To simplify the calculations, morphological dilation of the obstacles is employed to ensure that the physical distance between the path and obstacles exceeds a specified threshold, such as 2 mm, thereby imposing a hard constraint. Additionally, paths closer to the center of the safe window are preferred to achieve a soft constraint for increasing the distance from obstacles.

#### 2.3.6. Final Light Intensity Model

In designing puncture paths, clinicians often have varying preferences for different constraint conditions. To simulate these preferences, we propose an attenuation-based light intensity model that combines multiple normalized attenuation terms, each raised to a power determined by adjustable coefficients. The model is expressed as

(13) $$I=I_{\mathrm{obs}}\cdot {\left(A_{\mathrm{tra}}\right)}^{\alpha }\cdot {\left(A_{\mathrm{ref}}\right)}^{\beta }\cdot {\left(A_{\mathrm{vas}}\right)}^{\gamma }.$$

where $I_{\mathrm{obs}}$ is a binary indicator, taking values of 0 or 1, indicating whether the path is blocked by an obstacle. Further, 0 indicates an invalid path, and 1 indicates a valid path. $A_{\mathrm{tra}}$ represents the normalized tissue traversal depth by the puncture needle, ranging from 0 to 1, reflecting the light absorption of the tissue. $A_{\mathrm{ref}}$ represents the normalized cosine of the angle between the path and the skin, ranging from 0 to 1, reflecting the effect of light reflection. $A_{\mathrm{vas}}$ represents the normalized transmission absorption of medium-risk organs or tissues, such as blood vessels, ranging from 0 to 1. Here, ‘A’ denotes attenuation factors, each representing a specific constraint condition.

The coefficients α, β, and γ are adjustable parameters that reflect the clinicians’ preferences for the relative importance of each constraint condition. By using these coefficients as exponents, the model can adjust the impact of each attenuation factor more flexibly. For instance, a higher coefficient value increases the sensitivity of the overall light intensity to changes in the corresponding attenuation term. This approach ensures that the model comprehensively considers multiple factors, providing a holistic score to assist clinicians in selecting the optimal puncture path.

All attenuation terms ($A_{\mathrm{tra}}$, $A_{\mathrm{ref}}$, and $A_{\mathrm{vas}}$) and the obstacle indicator ($I_{\mathrm{obs}}$) are normalized variables, meaning they have been scaled to the range [0, 1] from their original values.

### 2.4. Implementation of the Optical Illumination-Inspired Path Planning Algorithm

**Figure 4:**
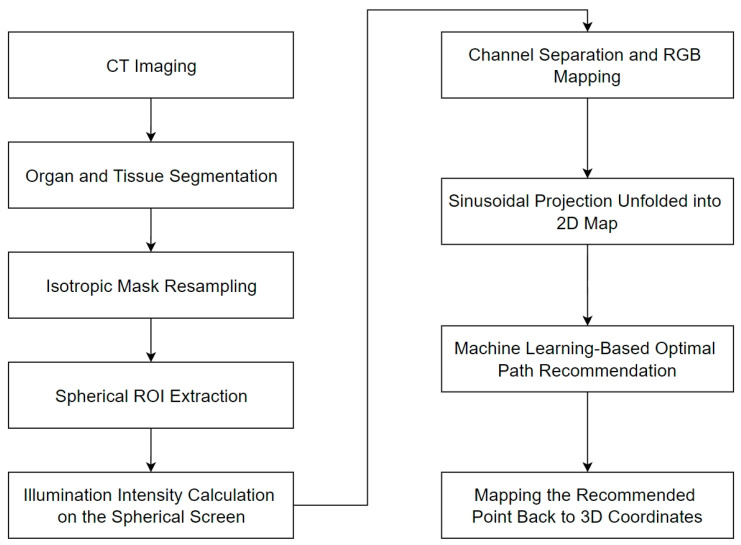
Flowchart of the proposed path recommendation system.

Figure 4 presents the complete workflow of the system, outlining each step involved in the recommendation process.

#### 2.4.1. Segmentation Mask Acquisition

We employ the trained SegResNet to obtain segmentation masks for organs and tissues, with the input being the CT image data of the patient. The output consists of segmentation masks for specific organs or tissues required for path planning, serving as the input for subsequent image processing steps.

#### 2.4.2. Mask Resampling

In clinical practice, CT images often exhibit anisotropic resolution, where the resolution varies across the x, y, and z axes. To simplify subsequent computations and facilitate geometric processing, we resample the masks after acquisition. We calculate the spacing parameters for each direction, select the smallest spacing as the target, and resample all directions to this uniform resolution. This ensures consistent resolution across all axes, thereby enhancing computational efficiency and accuracy.

#### 2.4.3. ROI Cropping

**Figure 5:**
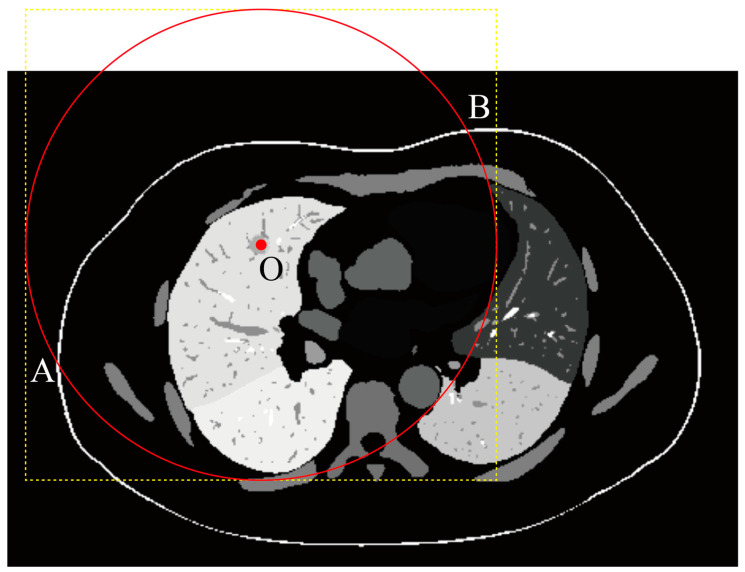
Workflow of the ROI extraction and zero-padding procedure. The red circle represents a sphere with a radius corresponding to the length of the biopsy needle (R). Points A and B mark the intersections of the red circle with the skin surface. The puncture target point O is first located, and an ROI boundary is defined by calculating with a radius equal to the biopsy needle length R. If the bounding box exceeds the image boundaries, zero-padding is applied to preserve symmetry. The yellow ROI is then cropped from the image. A spherical ROI with radius R is drawn, resulting in the red spherical ROI and shell. The region between the red spherical shell and the yellow bounding box is assigned a value of zero.

Following mask resampling, we proceed with ROI cropping to extract the pertinent area from the larger image volume. This step reduces data size, minimizes computational load, and eliminates irrelevant information. The cropping method involves drawing a sphere centered at the target point with a radius equal to the biopsy needle length and cropping along the bounding box of this sphere. To handle cases where the bounding box exceeds the CT image boundaries, we employ zero-padding. This padding does not introduce artificial data, and irrelevant regions are automatically excluded during computations. Additionally, we symmetrize the ROI to avoid asymmetrical calculations if the target is not at the center, thereby preventing issues related to varying distances and angle ranges. Figure 5 demonstrates the procedure described above.

#### 2.4.4. No-Skin Region Removal (Optional Step)

Based on the previously detected human skin surface, after performing spherical clipping, all points on the skin surface were connected to the target puncture point using line segments to define feasible regions. Regions where the needle length was insufficient were excluded, and the infeasible regions of the puncture needle located beneath the skin were directly marked as opaque areas to eliminate their interference in subsequent calculations.

#### 2.4.5. Intensity Calculation

A point light source emitting uniform light in all directions was placed at the target point, and the light intensity distribution on the spherical shell surface was calculated using the aforementioned model. First, a minimum distance threshold (e.g., 2 mm) was set for obstacles, and voxel dilation was performed on the obstacles to ensure that their influence range was adequately represented. In the optical property settings, obstacles (such as non-target lung lobes, the heart, the liver, the spleen, the pancreas, bones, the trachea, and major blood vessels, which are high-risk organs or tissues) were designated as opaque regions, while the target tissue (e.g., lung nodules) and background voxels were set as fully transparent. For passable, non-hazardous tissues (e.g., certain soft tissues), a higher transparency (absorption coefficient of 0.02 or other appropriate values) was assigned to simulate their attenuation effect during light propagation. For pulmonary blood vessels, a moderate transparency (absorption coefficient of 0.1 or other appropriate values) was assigned to reflect the influence of vessel thickness or their stacking quantity on light intensity. To achieve efficient computation, various optimization techniques were employed, including early ray termination (terminating ray calculations when full occlusion was reached), empty space skipping (skipping empty data regions to reduce computational load), sample step size reduction (improving computational accuracy by reducing sampling step size), gradient precomputation (precomputing gradients to accelerate lighting calculations), and GPU acceleration and multithreading, significantly enhancing computational speed.

**Figure 6:**
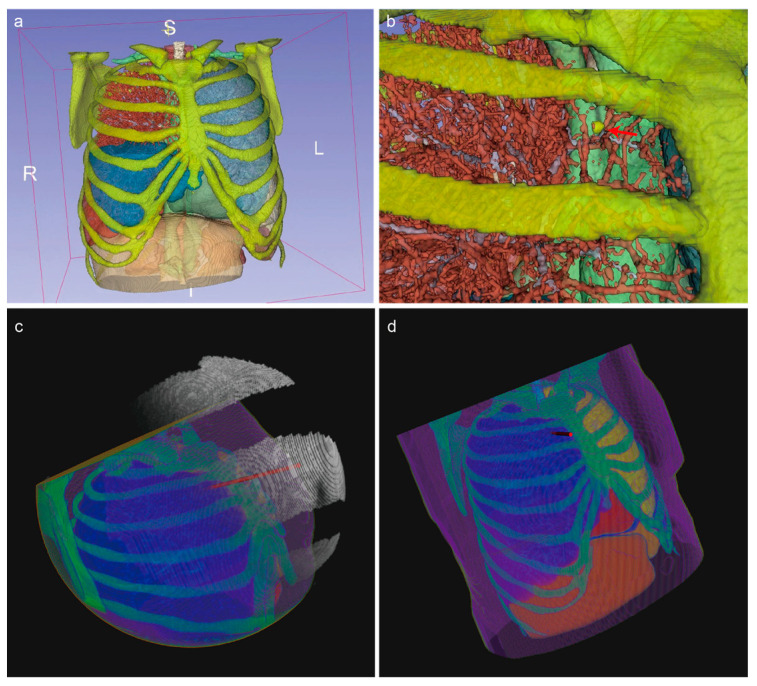
Visualization of segmentation masks, illumination intensity distribution, and puncture path mapping. (**a**) Three-dimensional rendering of organ and tissue segmentation masks, with skin and body tissues hidden for clarity. (**b**) Magnified view of the puncture target area with the target lung lobe hidden. The red arrow indicates the puncture target point. (**c**) Typical illumination intensity distribution result. The human body mask is spherical due to ROI cropping, with flat cuts and zero-padding beyond the CT image boundaries. The spherical screen displays the light intensity distribution as a thin point cloud, enabling the selection of the optimal puncture entry point. (**d**) Three-dimensional mapping of the selected puncture path. The red cylinder represents the simulated puncture needle path.

Figure 6a presents a 3D rendering of the organ and tissue segmentation masks for a sample case. To enhance visibility, the rendering excludes the skin and body tissues. Figure 6b is a magnified view of the target area with the target lung lobe hidden for clarity. The red arrow indicates the puncture target point. Figure 6c illustrates a typical result of the illumination intensity distribution described earlier. The human body mask appears spherical due to ROI cropping. Where the ROI intersects the CT image boundaries, the cuts are flat, and zero-padding is applied beyond these boundaries. The white image shows the light intensity distribution on the spherical screen, represented as a thin point cloud. Users can select an appropriate puncture entry point based on the light intensity distribution and the size of the illuminated regions. Figure 6d shows the result of mapping the selected puncture path back into 3D space. The red cylinder represents the simulated puncture needle path.

#### 2.4.6. Optimal Path Recommendation

a. **Illumination Map Generation and RGB Projection**

As illustrated in Figure 6c, all non-black candidate regions are potential starting points for puncture. Regions with higher intensity values are considered more suitable, while areas farther from black regions (representing obstacles) are preferred. Accordingly, the center of a highlighted, large-area region is recommended as the puncture target. The intensity values in these regions are influenced by three factors: skin angle, body thickness, and pulmonary vessels. Optimizing these parameters can be complex, so we developed a machine learning-based solution for path recommendation.

First, the centroid of the labeled lung nodule voxels was calculated as the biopsy target point. Using our illumination computation algorithm, we determined the light intensity distribution over the surface of a sphere surrounding the target. To improve the effectiveness of the machine learning modeling, we separately computed three attenuation terms, $A_{\mathrm{tra}}$, $A_{\mathrm{ref}}$, and $A_{\mathrm{vas}}$, and an obstacle indicator, ($I_{\mathrm{obs}}$). Since $I_{\mathrm{obs}}$ is binary (0 or 1), it was multiplied directly with $A_{\mathrm{ref}}$ to reduce the original four datasets to three. These datasets were then mapped to the red, green, and blue (RGB) channels, respectively, and projected onto the spherical surface using a sinusoidal projection.

**Figure 7:**
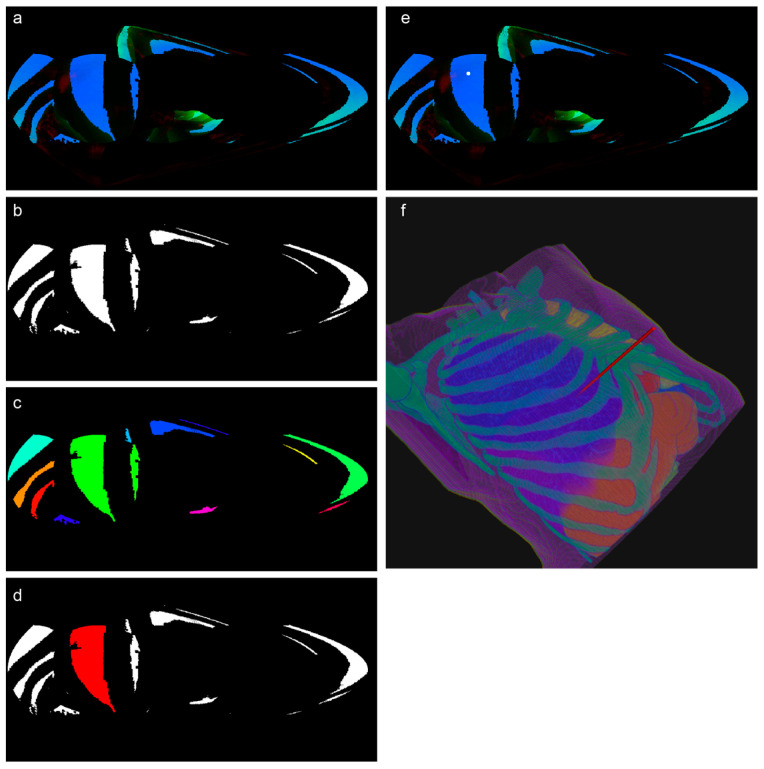
From illumination map to puncture path: visual biopsy trajectory planning. (**a**) RGB Sinusoidal Projection: Illumination results (attenuation terms and obstacle indicator) mapped to RGB channels and projected onto a 2D planar image using sinusoidal projection, visualizing feasible puncture regions while preserving area ratios. In the RGB projection, the blue, red, and green channels, respectively, represent the intensity parameters for ‘ref’, ‘vas’, and ‘trans’ terms. (**b**) Initial Region Segmentation: Low-risk regions are segmented by applying statistical thresholds to the RGB values, determining the RGB value ranges for safe regions based on the statistical distribution of the RGB channels, thus identifying potential areas of interest. (**c**) Filtered Safe Regions: Filtered safe regions were obtained after setting a size threshold based on clinical experience to remove smaller candidate regions. (**d**) Expert Annotation: Expert annotation by a thoracic surgeon who selected a recommended needle insertion region based on their clinical experience. (**e**) Final Puncture Point: Final puncture point determined by expert annotation or machine learning prediction and refined to a single pixel via iterative erosion (indicated by a white dot). (**f**) Biopsy Trajectory: 2D puncture point reverse-mapped to 3D CT space, visualizing the calculated biopsy needle trajectory (red cylinder).

The sinusoidal projection, an equal-area projection method, preserves the relative area sizes and provides an intuitive visualization of the feasible puncture regions. As CT scanning beds are typically positioned below the patient, we chose the ventral side (anterior) of the ROI as the projection center. The RGB channels were combined into a single image, as shown in Figure 7a.

b. **Region Segmentation and Expert Annotation**

The segmentation of low-risk regions was guided by the statistical distribution of the RGB values across the dataset. Thresholds were applied to the RGB channels to identify the regions of interest, effectively dividing the images into distinct segments. Using these thresholds, safe regions were extracted, segmented into discrete connected components, and filtered to exclude small regions below a set size threshold (Figure 7b,c).

To incorporate clinical expertise, an experienced thoracic surgeon reviewed the segmented regions and annotated the preferred puncture points on the projected images (Figure 7d).

c. **Feature Extraction and Dataset Preparation**

Features were then extracted from each connected region using the regionprops function from the scikit-learn library (version 1.6.1). Using scikit-image (version 0.24.0), a total of 50 categories of features were computed, capturing geometric, physical, and intensity-based properties such as area, axis lengths, bounding box dimensions, centroids, shape descriptors, pixel intensity metrics, moments, and statistical summaries.

A total of 250 annotated projected images (LUNA16) were prepared, each containing multiple connected regions. The annotated dataset was divided into training, validation, and test subsets in a 7:1:2 ratio. To ensure no data leakage, all connected regions from a single image were assigned to the same subset. This resulted in 175 images and 1486 regions for training, 25 images and 215 regions for validation, and 50 images and 386 regions for testing. Each region was labeled with a binary variable indicating whether it was selected by the surgeon.

Columns with missing values, introduced during feature extraction, were removed. Significant class imbalance was observed in the dataset, with fewer positive (surgeon-selected) regions compared to negatives. To address this, the Synthetic Minority Oversampling Technique (SMOTE) was applied to the training data, ensuring balanced representation of both classes.

d. **Machine Learning Model Training and Evaluation for Region Selection**

To evaluate the region selection task, we employed and compared machine learning models and multi-layer perceptron (MLP) neural networks.

Four traditional machine learning models were trained: random forest, XGBoost, LightGBM, and CatBoost, each configured to handle class imbalance. The key parameter settings for each model are described below, with other parameters kept at their default values as provided by the scikit-learn (version 1.6.1) and CatBoost libraries (version 1.2.7) unless otherwise specified.

- Random Forest: The random forest model was configured with 100 trees to ensure robust performance. The maximum depth of each tree was limited to 10 levels to prevent overfitting. To address class imbalance, the class_weight parameter was set to “balanced”, which automatically adjusts weights inversely proportional to class frequencies.
- XGBoost: The XGBoost model was trained using 100 boosting rounds. The maximum depth of each tree was set to 6 levels. To handle class imbalance, the scale_pos_weight parameter was used, which was set to the ratio of negative to positive samples in the resampled training data. The evaluation metric was set to log loss for binary classification.
- LightGBM: The LightGBM model was configured with 100 boosting rounds. To address class imbalance, the class_weight parameter was set to “balanced”.
- CatBoost: class imbalance was addressed using the class_weights parameter, with weights explicitly set to [1, ratio of negative to positive samples in resampled training data] to balance class influence.

In addition to these traditional machine learning models, we also investigated the performance of MLP classifiers with varying architectural configurations to assess the impact of network complexity. Four distinct MLP models were designed and implemented:

- MLP Simple: Serving as a baseline shallow network, this model comprised a single hidden layer of 100 neurons. This architecture was designed to establish a performance reference point against which more complex models could be compared.
- MLP Deep: To investigate the influence of network depth, we implemented a two-hidden-layer MLP. The architecture consisted of a first hidden layer with 100 neurons, followed by a subsequent layer of 50 neurons. This configuration aimed to determine whether increased depth, while maintaining a constrained number of neurons per layer, could enhance feature representation and, consequently, model performance.
- MLP Wide: Complementing the exploration of depth, the “MLP Wide” model assessed the impact of network width. This architecture utilized a single hidden layer, but with an expanded capacity of 200 neurons. This design sought to evaluate whether a wider hidden layer, affording increased representational capacity within a single layer, would yield performance advantages.
- MLP Complex: A more elaborate architecture, “MLP Complex”, was implemented to integrate both increased depth and width. This model incorporated three hidden layers with a progressively refined neuron count: 150, 75, and 30 neurons in successive layers. This configuration was designed to examine the potential benefits of a more intricate network structure capable of hierarchical feature learning and capturing potentially nuanced relationships within the data.

Across all MLP models, the ReLU activation function was employed within hidden layers, and the Adam optimizer was utilized for training with a maximum of 300 iterations.

Additionally, we invited a junior doctor to serve as a control. He was asked to select connected regions on the RGB projection map, map them to 3D puncture paths, and then compare these paths with the annotations provided by a senior doctor. The feasibility of paths planned by the junior doctor was subsequently evaluated.

e. **Final Path Recommendation Algorithm Testing**

The path planning algorithm was tested on the 50 images in the test set. For each sample, the puncture point was determined using the best-performing machine learning model. The total runtime for generating the puncture point was recorded for each case. Subsequently, the feasibility of each path was assessed by the thoracic surgeon, who also provided a satisfaction score based on personal preferences. The satisfaction scoring system was defined by factors such as ease of access, safety, and alignment with clinical objectives.

During inference, the model assigned probabilities to each region, classifying the region with the highest probability as positive and the others as negative. Model performance was evaluated using standard metrics:

- Area Under the Receiver Operating Characteristic Curve (AUC-ROC): this assesses the overall classification ability, balancing sensitivity and specificity.
- Precision, Recall, and F1-score: these measure the quality of positive predictions and account for class imbalance.
- Specificity: this evaluates the ability to accurately identify negative regions.
- Sensitivity: this evaluates the ability to accurately identify positive regions.
- Confusion Matrix Components: this provides a detailed breakdown of the prediction outcomes, including TP, FP, TN, and FN.

It is important to note that while these symbols are similar to those used in the segmentation evaluation section, they pertain to the classification task in this context.

The final puncture point was determined based on either the annotations of the surgeon or the predictions of the machine learning model. For the selected target region, iterative erosion was applied until a single pixel remained, representing the puncture point (indicated as a white dot in Figure 7e). This 2D point was then reverse-mapped to the original 3D space to calculate the biopsy trajectory (Figure 7f, where the red cylinder represents the needle path).

## 3. Results

### 3.1. Segmentation Model Performance

#### 3.1.1. Model Training and Setup

We compared three high-resolution segmentation architectures (SegResNet, 3D-UNet, and UNETR) within the nnU-Net framework using our annotated LUNA16 dataset. Our pipeline uses a coarse-to-fine strategy that begins with a low-resolution ROI extractor and is followed by high-resolution segmentation. All models were trained with identical parameters (see Methods for details).

#### 3.1.2. Quantitative Segmentation Accuracy Comparison

Table 1, Table 2 and Table 3 present the quantitative segmentation accuracy of SegResNet, 3D-UNet, and UNETR, respectively, across various anatomical structures. A comparative analysis of these results reveals performance variations among the models.

Across key organs, SegResNet demonstrates a numerically higher DSC. For instance, SegResNet achieves DSC scores of 0.9684 for the heart, 0.9830 for the liver, and 0.9691 for the spleen. Similarly, for bone segmentation (excluding the spine), SegResNet attains a DSC of 0.9144. In comparison, while 3D-UNet and UNETR also show strong performance on these organs, their DSC values are generally slightly lower (refer to Table 1, Table 2 and Table 3 for specific values).

Furthermore, the results indicate differences in vascular segmentation accuracy. For the pulmonary arteries and veins, both 3D-UNet and SegResNet demonstrate comparable segmentation performance (refer to Table 1 and Table 2 for DSC values). However, both 3D-UNet and SegResNet achieve numerically higher DSC scores for pulmonary arteries and veins when compared with UNETR (Table 3), indicating superior performance over UNETR in segmenting these particular vascular structures. For example, for pulmonary arteries, 3D-UNet and SegResNet achieve DSC scores of approximately 0.88–0.89, while the DSC of UNETR is considerably lower at 0.6460. Similarly, for pulmonary veins, 3D-UNet and SegResNet achieve DSC scores around 0.85–0.86, compared to the DSC of UNETR at 0.7635.

For lung nodule segmentation, all models exhibit lower DSC scores compared with other anatomical structures, suggesting that this task is more challenging. While achieving modest DSC values across the board, the DSC of SegResNet for nodule segmentation (0.5328) is numerically lower than that of 3D-UNet (0.5756), but remains higher than the DSC of UNETR (0.3675). This indicates that while the performance of SegResNet on lung nodules is not as strong as that on other organs, it still outperforms UNETR in this specific, challenging segmentation task, although it lags slightly behind 3D-UNet.

These quantitative comparisons indicate that SegResNet, which incorporates residual connections in its architecture, tends to yield numerically superior or comparable segmentation accuracy across the evaluated anatomical structures when compared to 3D-UNet and UNETR. The following sections will explore the implications of these segmentation performance differences for subsequent path planning tasks.

#### 3.1.3. Ablation Analysis of Segmentation Experiments

To comprehensively assess segmentation accuracy and system efficiency, we conducted ablation experiments focusing on different backbone modules within our segmentation models.

The experiments were performed under controlled conditions on a local server equipped with two Intel Xeon Gold 6133 CPUs (2.5 GHz), four NVIDIA RTX 4090 GPUs (24 GB each), and 128 GB of RAM. For consistency, all inference tests were run on a single GPU with the CPU thread count limited to 3. The runtime environment included CUDA 11.8, Python 3.11, and PyTorch 2.4.0. The ablation experiment was tested on the test dataset comprising 50 CT files. On average, these files contained 316 ± 31 slices along the *z*-axis.

As shown in Table 4, the SegResNet model (U-Net + Res Block) achieved the highest segmentation performance, with an average DSC of 0.9122. This improvement over the 3D U-Net model (U-Net + Base Conv) suggests that the residual blocks enhance feature extraction capabilities. Conversely, although UNETR (U-Net + Transformer Block) incorporates the theoretically more powerful Transformer block, it did not perform optimally for our specific task.

In terms of model efficiency, the 3D U-Net model (U-Net + Base Conv) was the simplest, resulting in the most efficient inference time and GPU memory consumption. The SegResNet model (U-Net + Res Block), which incorporated residual structures, had an average inference time of 63.601 s and a GPU memory usage of 4321 MB, slightly higher than the 3D U-Net model. In contrast, UNETR (U-Net + Transformer Block) suffered from the high computational cost of Transformer blocks, resulting in no advantage in inference speed or GPU efficiency.

Based on the superior segmentation performance and balanced resource consumption of SegResNet, we selected it as our segmentation model.

#### 3.1.4. Segmentation Robustness Analysis

The previous experiments were trained and validated on the LUNA16 dataset. To assess the generalization and robustness of our segmentation model, we selected the best-performing SegResNet model for testing on both publicly available and private datasets. The public datasets used were the Tianchi Lung Nodule dataset and the LNDb dataset, while the private dataset consisted of clinical data provided by partner hospitals. The segmentation results obtained using the SegResNet model are presented in Table 5.

In Table 5, segmentation performance on the publicly available Tianchi and LNDb datasets is slightly lower than on the LUNA16 training dataset—an expected outcome that underscores the commendable generalization ability of the model. Although the public datasets exhibit moderate variability in data distribution, the private clinical dataset shows more pronounced differences due to variations in CT equipment and specialized acquisition parameters, which leads to a further drop in performance. This anticipated decline emphasizes that practical deployment will benefit from model fine-tuning or domain adaptation to accommodate diverse clinical environments. Nonetheless, our segmentation model achieved an impressive average DSC score of 0.8563, affirming its robust overall performance.

### 3.2. Path Planning and Recommendation Algorithm Performance

#### 3.2.1. Quantitative Evaluation of Path Planning and Machine Learning-Based Recommendation Algorithms

To develop and test our path planning and recommendation algorithms, we processed the in-house annotated mask data from the LUNA16 dataset. For each sample, an experienced surgeon designated the puncture target point—typically the physical center of a nodule—and fixed the biopsy needle length at 153 mm. We then applied our proposed path planning algorithm to compute the virtual illumination distribution for each sample and mapped the results into sinusoidal projection RGB images. Next, connected regions were extracted using the method described in Methods 2.4.6. The experienced surgeon subsequently annotated these regions by selecting his preferred puncture paths. Finally, machine learning models were trained to mimic his preferences, thereby implementing our proposed “surgeon behavior mimicking path recommendation approach”.

We evaluated four machine learning models—random forest, XGBoost, LightGBM, and CatBoost—using a dataset comprising over 2000 labeled regions across 250 annotated projected images. Each model was trained using a balanced dataset, created through SMOTE, to address the class imbalance.

The models were evaluated on the test set of 50 images using a variety of performance metrics, including AUC-ROC, precision, recall, F1-score, specificity, and confusion matrix components, which provide a comprehensive understanding of their effectiveness. The performance results are presented in Table 6. Although CatBoost achieved the highest AUC-ROC of 0.951, random forest exhibited a commendable balance across metrics, with an AUC-ROC of 0.948, a high recall of 0.766, and a solid precision of 0.706, culminating in an overall F1-score of 0.735. Its robust specificity (0.956) further indicates reliable discrimination between the positive and negative regions.

In addition to accuracy, inference speed is critical for clinical applications. As presented in Table 7, the random forest model recorded the lowest average time cost at 0.072 ± 0.022 s, outperforming XGBoost (0.081 ± 0.022 s), LightGBM (0.106 ± 0.028 s), and CatBoost (0.082 ± 0.028 s). This rapid processing capability, combined with its balanced predictive performance, supports the rationale for integrating the random forest model into the pipeline.

Overall, while each model offers valuable performance, the optimal balance of accuracy and computational efficiency of the random forest model makes it particularly suitable for real-time clinical deployment. Nonetheless, further model fine-tuning or domain adaptation remains essential to address data distribution variations and enhance performance across diverse clinical environments.

#### 3.2.2. Evaluation of Biopsy Path Parameters and Clinical Feasibility

In addition, we evaluated the key clinical parameters of the planned paths, including the depth of needle insertion, the angle relative to the skin, and the minimum distance to critical organs (Table 7). For example, the random forest model produced an average insertion depth of 70.774 ± 29.320 mm, an average angle of 63.353 ± 11.216°, and an average minimum distance of 3.326 ± 3.512 mm, which are very close to those of the surgeon-selected paths (ground truth: 71.640 ± 31.556 mm for depth, 62.696 ± 11.794° for angle, and 3.142 ± 3.590 mm for distance). This strong numerical agreement further validates the feasibility of our machine learning approach.

Given the performance of the random forest model, we constructed a path planning pipeline comprising the following steps: (1) segmentation mask processing, (2) virtual illumination calculation, (3) sinusoidal projection to RGB images, (4) connected region segmentation, (5) feature extraction, (6) random forest inference to select the optimal entry region, and (7) reverse computation of the 3D entry point coordinates to construct the needle trajectory.

We assessed the performance of the path recommendation algorithm using 50 cases from the test set, examining its operation from segmentation mask processing to needle trajectory generation when integrated with the random forest model. On a PC with an Intel i7-12700 CPU, 32GB RAM, and an NVIDIA GeForce RTX 3050 GPU, the algorithm generated biopsy trajectories with an average runtime of 1.905 s (standard deviation: 0.089 s), with a minimum of 1.770 s and a maximum of 2.160 s, demonstrating both efficiency and consistency in generating paths.

To evaluate the clinical feasibility of the recommendations of the algorithm, the experienced thoracic surgeon assessed the generated paths for safety and ease of access. All 50 recommended paths were considered feasible, and the surgeon provided a satisfaction rating for each case based on criteria such as alignment with clinical goals, safety, and accessibility. The majority of the recommendations (78%, 39/50) were rated as fully satisfactory, while the remaining 22% (11/50) were rated as moderately satisfactory. Notably, there were no instances where the paths were deemed unsatisfactory (0/50).

These results suggest that the algorithm is capable of not only generating feasible biopsy paths in a clinically relevant timeframe, but also aligning with surgeon preferences, supporting its potential for use in real-world clinical settings.

#### 3.2.3. Ablation and Comparative Study

To evaluate our machine learning-driven approach, we conducted an ablation and comparative study with a twofold strategy. First, we assessed the efficacy of the neural network architectures by replacing traditional machine learning classifiers with four distinct MLP configurations to determine if these neural network methods offered superior performance. Second, to isolate the impact of automated entry region selection, we performed an ablation by removing step (6), the random forest inference for optimal entry region selection, from our seven-step “segmentation mask to needle trajectory” pipeline. In this modified configuration, a junior surgeon manually selected the entry region.

We quantitatively evaluated the accuracy (Table 6), path parameters, and computational efficiency (Table 7) of these varied approaches. Independent timing of the decomposed pipeline steps revealed that generating RGB sinusoidal projection images required an average of 1.758 ± 0.061 s. MLP-based methods demonstrated statistically comparable accuracy to traditional machine learning classifiers (see Table 6), albeit with subtle variations in specific metrics. However, manual selection by the junior surgeon resulted in significantly decreased classification performance, particularly evident in the reduced recall (0.500) and F1-score (0.569). The computational speed of the MLP-based methods (averaging 0.053–0.055 s) was similar to that of the machine learning models. In contrast, manual selection was significantly slower, requiring 1.968 ± 0.802 s on average for the junior surgeon. While our approach significantly simplified the inherently complex task of 3D spatial reasoning and visualization—a process that internally, in unassisted settings, takes approximately 15 s—manual human cognitive processing and operation still proved to be substantially slower. Manual selection also led to decreased accuracy.

Importantly, the path parameters (average depth, angle, and distance) for MLP-based and manual methods remained clinically comparable to those achieved with the machine learning models and ground truth. This suggests that while manual selection and MLPs might impact region prediction accuracy, the fundamental path characteristics are preserved. The clinical evaluation, demonstrating a 100% (50/50) pass rate and a 66.0% (33/50) excellent rate for junior manually selected paths, provides compelling evidence for the overall reliability and clinical translatability of our proposed approach, even in scenarios incorporating manual surgeon input.

These findings highlight the robustness and adaptability of our machine learning-driven path planning system, demonstrating its potential for integration into clinical workflows.

## 4. Discussion

### 4.1. Key Findings

This study successfully developed and validated a novel, rapid, and clinically feasible method for robot-assisted percutaneous puncture, with three key findings: **(1) ultra-fast path computation**, with the entire pipeline using a random forest algorithm running in 1.905 s; **(2) strong clinical feasibility**, evidenced by a thoracic surgeon rating 78% of algorithm paths as fully satisfactory and 100% as clinically feasible; and **(3) high-performance anatomical segmentation** suitable for lung puncture procedures. Through a detailed dataset of segmentation labels, the models trained with SegResNet achieved an average DSC of 0.9122. Furthermore, ablation studies showed framework flexibility, with MLP architectures achieving path recommendation accuracy comparable to that of traditional machine learning models. Additionally, manual entry region selection by a junior surgeon significantly reduced region prediction accuracy and increased procedure time, underscoring the value of automated and rapid path planning.

Ultra-fast computation times were achieved through the innovative application of optical principles and efficient GPU parallel processing. By reformulating needle path planning as a light propagation simulation, computationally intensive geometric calculations were bypassed. Simulating light absorption and reflection—mirroring needle–tissue interactions and safety constraints—enabled the rapid generation of candidate puncture sites. This approach leverages the inherent straight-line propagation of light, reflecting rigid needle trajectories, and its omnidirectional emission to effectively explore the 3D search space for optimal entry points.

Furthermore, GPU acceleration via the NVIDIA engine facilitated highly efficient ray tracing, dramatically reducing computational overhead. Performance optimization involved early ray termination, empty space skipping, sample step size reduction, and gradient precomputation, which, combined with GPU acceleration and multithreading, significantly enhanced the computational speed.

Another significant factor in rapid computation is the successful integration of vascular modeling into our simulated illumination model. Unlike critical organs, the vasculature has partial traversability: larger vessels are strictly avoided, while smaller, sparse networks may be permissible. Traditionally, vasculature processing requires calculating vessel trajectories, diameters, and path intersections, demanding substantial computation. Our approach circumvents this by assigning absorption coefficients to vessels, integrating them into a unified algorithmic model without separate processing. Furthermore, 2D mapping to RGB channels isolated vascular information, enabling machine learning to mimic surgeon preferences, obviating intricate parameter adjustments and enhancing operational speed and clinical applicability.

The strong clinical feasibility likely stems from the intuitive grayscale map on a virtual sphere shell around the puncture target, visually representing puncture site desirability. Accumulated absorption (simulating needle depth/risk) and diffuse reflection (modeling needle angle constraints) were comprehensively encoded. This intuitive visual output may facilitate rapid assessment, aligning with experienced surgeons’ cognitive workflows and contributing to high clinical satisfaction. The grayscale map likely simplifies reconstructing 3D anatomy from 2D CT images, potentially reducing cognitive load and enhancing surgeon confidence. Additionally, RGB channel mapping minimizes intricate parameter adjustments, enhancing operational efficiency and clinical applicability.

While primarily focused on developing an advanced path planning technique, this study systematically evaluated three high-resolution neural network architectures—SegResNet, 3D-UNet, and UNETR—trained on our annotated LUNA16 dataset with identical parameters within the nnU-Net framework to ensure robust anatomical segmentation for accurate input data. Quantitative analysis (Table 1, Table 2 and Table 3) revealed that SegResNet consistently outperformed the others in terms of DSC across key organs (heart: 0.9684, liver: 0.9830, spleen: 0.9691), while 3D-UNet and UNETR, though still robust, showed slightly lower metrics. In vascular segmentation, SegResNet and 3D-UNet demonstrated comparable performance (DSC ~0.88–0.89 for pulmonary arteries and 0.85–0.86 for pulmonary veins), significantly outperforming UNETR (DSC: 0.6460 and 0.7635, respectively), highlighting the effectiveness of convolutional architectures for these structures. Although lung nodule segmentation proved more challenging (SegResNet: 0.5328, 3D-UNet: 0.5756, UNETR: 0.3675), SegResNet still outperformed the Transformer-based UNETR, despite slightly lagging behind 3D-UNet. These variations suggest that the residual connections of SegResNet enhance feature extraction, yielding superior or comparable accuracy across most anatomical structures, while the Transformer module of UNETR may struggle with fine-grained local details essential for precise segmentation. Ultimately, SegResNet was chosen as our definitive segmentation model due to its consistently excellent performance and optimal balance of accuracy and resource consumption (inference time and GPU memory usage are modestly higher than 3D-UNet, yet substantially lower than UNETR), ensuring overall efficacy for path planning input generation.

### 4.2. Comparative Analysis with Existing Methods

This study presents a path planning method that uniquely combines speed, simplified vascular modeling, and clinical feasibility, positioning our advancements within the field through systematic comparison. Table 8 provides a comparative summary of our method in relation to relevant prior research.

#### 4.2.1. Computation Speed

The ultra-fast computation time of our method significantly contrasts with many existing rigid needle path planning algorithms that prioritize accuracy or comprehensive modeling, often incurring substantial computational overhead and longer processing times. For instance, Zhang et al. (2024) [25] reported algorithm runtimes exceeding 40 min, with constraint evaluation being the most time-consuming. Similarly, Liu et al. (2023) [5] noted constraint calculations pushing system execution times beyond 4000 s. Yang et al. (2024) [17] documented an average planning time of approximately 3 min (179.11 s), with over 100 s for entry point selection. Song et al. (2024) [8] and Li et al. (2023) [22] reported automatic planning times of around 4 min and 41.4 s, respectively. Baegert (2007) [33] described planning times from 30 s (no subdivisions) to 2 min (three subdivisions), while Dong et al. (2022) [26] implied that iterative Non-dominated Sorting Genetic Algorithm (NSGA)-III methods require multiple runs. Additionally, Liu et al. (2023) [5] indicated that constraint evaluation dominates the computational burden, with a total system runtime ranging from 2045.3 to 4518.3 s. Xie et al. (2023) [41] mentioned that single CT image processing takes about 5 s, and Too et al. (2024) [6] reported complete process times under 5 min. Monfaredi et al. (2024) [45] noted that while brute-force search is computationally intensive, GPU acceleration and tailored optimization can reduce computation times from minutes to seconds.

In contrast, our approach achieves path generation in under two seconds, with organ segmentation taking approximately one minute. Unlike heuristic and optimization-based methods typically requiring tens of seconds to minutes per plan, our method efficiently integrates GPU-accelerated ray tracing with optical simulation and machine learning-based recommendation. For example, Zhengshuai Wang et al. (2024) [39] reported an average puncture planning time of 35 s using a bounding box and Pareto optimization. Our method consistently delivers rapid computation in under two seconds, a substantial improvement.

It is important to note that Min Luo et al. (2022) [16] reported optimal puncture path computation times under 200 ms; however, their timing method differed, with some path planning steps occurring in a 5 to 20 min preprocessing stage.

Overall, our approach demonstrates a significant speed advantage over methods relying on extensive preprocessing, complex constraint evaluation, or iterative multi-objective optimization, making our solution highly adaptable to dynamic clinical scenarios requiring fast decision-making.

#### 4.2.2. Accuracy and Performance

Evaluating the accuracy and performance of path planning algorithms is complex due to the diverse metrics across studies, necessitating cautious direct numerical comparisons. This section contextualizes the performance of our algorithm, acknowledging the limitations of comparing disparate metrics.

The accuracy of our algorithm in path recommendation, primarily assessed by its ability to predict the preferences of a surgeon, achieved an AUC-ROC of 0.948. This, along with precision, recall, and F1-score, indicates robust predictive capability within machine learning methods aligning with expert clinical decision-making in path planning. Note that AUC-ROC, focused on ranking path options by the preference of a surgeon, differs fundamentally from metrics in studies on geometric deviation or clinical feasibility.

Our random forest pipeline recommended paths with an average depth of 70.774 mm, angle of 63.353°, and distance to dangerous organs of 3.326 mm. In comparison, the liver puncture study of Dong et al. (2022) [26] reported average dangerous distances of 7.52–28.75 mm, liver surface angles of 34.53–64.02°, and intrahepatic depths of 21.87–62.53 mm; however, liver anatomy differs significantly from the lung, limiting direct comparison. The liver ablation algorithm of Li et al. (2023) [22] reported a mean puncture angle of 3.6° (range 0–18.3°), a depth of 42.3–98.8 mm, and an average ablation efficiency of 60.19%; their research context also differs from our lung path planning.

The clinical feasibility of our method is supported by the positive evaluation from an experienced thoracic surgeon, with 78% of recommended paths rated as fully satisfactory and 100% deemed clinically feasible, supporting its clinical application potential. Wang et al. (2024) [2] found a 100% pass rate (61.11% excellent) for 90 paths in 18 cases, with qualitative evaluations showing 82.22% considered reasonable and clinicians favoring automatic planning in nine and seven cases, respectively, noting higher efficiency and practical value for less experienced clinicians. The prospective study of Yang et al. (2024) [17] on 25 lung puncture cases showed feasible and relatively easy paths for most, indicating good versatility and operability, with only one infeasible and three difficult paths noted. The retrospective study of Too et al. (2024) [6] (150 cases) showed that 82% of paths matched actual biopsies (angle deviation < 5°), with 85.3% accepted as safe. The clinical validity study of Song et al. (2024) [8] found that 50% of automatically planned paths were “excellent” or “acceptable”, rising to 78% after interactive adjustment; clinicians considered 78% of automatically planned paths valid and aligned with clinical procedures, noting better planning efficiency than manual operation. The liver ablation algorithm study of Li et al. (2023) [22] reported that two experienced clinicians found that all planned paths clinically needed and aligned with their operating habits. The evaluation of Min Luo et al. (2022) [22] by physicians with varying experience showed high satisfaction, indicating the clinical acceptability of their radiofrequency ablation (RFA) plan. The blind evaluation of Qi Liu et al. (2023) [5] by experienced interventional radiologists found all automatically planned paths feasible in Dataset 1 (5 patients) and 87.5% clinically acceptable in Datasets 2 and 3 (24 patients). The retrospective study of Ling He et al. (2023) [44] on the OLPCT dataset found algorithm paths clinically feasible in all cases, with 23 of 25 cases deemed feasible in a prospective study on the NLPCT dataset.

While direct numerical comparison of our AUC-ROC to deviation or feasibility metrics in other studies is statistically invalid due to their differing nature, our results demonstrate clinically relevant accuracy. Our AUC-ROC indicates a strong ability to predict the preference of a surgeon, which is crucial for the clinical usability of a path planning tool designed to assist, not replace, expert judgment. Furthermore, a positive clinical feasibility assessment by a surgeon supports the practical applicability and clinical relevance of our approach.

The performance of our machine learning model, like any data-driven approach, inherently depends on the quality and representativeness of the training data regarding the preferences of the surgeon. However, the robust AUC-ROC, combined with positive clinical feasibility feedback, suggests that our method achieves a competitive and clinically meaningful accuracy level within its intended application of predicting and recommending puncture paths preferred by a surgeon.

#### 4.2.3. Novel Vascular Modeling

Our simplified modeling of the vasculature, based on assigning absorption coefficients within a simulated optical environment, contrasts with conventional techniques for vascular modeling in path planning. Baegert et al. (2007) [33], Dong et al. (2022) [26], Too et al. (2024) [6], Song et al. (2024) [8], Zhang et al. (2024) [25], Liu et al. (2023) [5], Xie et al. (2023) [5], and He et al. (2023) [44] employ “hard-constraint” approaches, treating vessels as absolute or near-absolute exclusion zones with varying implementations. For instance, Baegert (2007) [33] used vessels as obstacles in visibility testing. Dong et al. (2022) [26] and Li et al. (2023) [15] used morphological expansion for vessel avoidance. Too et al. (2024) [6] penalized paths intersecting vessels of diameters greater than 2mm. Song et al. (2024) [8], Zhang et al. (2024) [25], Liu et al. (2023) [5], and He et al. (2023) [44] implemented hard constraints to completely avoid vessels. Xie et al. (2023) [21] focused on avoiding “key vessels” through 3D model observation and grayscale information, without explicit quantification of vessel distance. Li et al. (2023) [15] also treated vessels of diameter larger than 3 mm as constraint regions, applying a 1 mm morphological expansion to further ensure avoidance. Monfaredi et al. (2024) [43] highlighted the general trend of considering vessels as key structures for avoidance in path planning for various interventional tools, including steerable needles and concentric tube robots. Yang et al. (2024) [17] similarly incorporated vessel avoidance in preprocessing (vessel segmentation for anatomical context) and path evaluation (penalizing higher vessel density within a path’s vicinity as a risk indicator) stages. Wang et al. (2024) [38] also treated vessels as vital organs for avoidance as a hard constraint, using a marching cubes algorithm to extract vessel surfaces and mapping them to a bounding box to define infeasible regions for needle insertion, ensuring paths avoid contact with vessels. In contrast, our approach offers a more nuanced perspective on risk to the vasculature by assigning coefficients of absorption, implicitly modeling varying degrees of traversability based on vessel size and density, potentially allowing paths to permissibly traverse smaller vessels, aligning with the clinical understanding of acceptable puncture through smaller vessels. None of the cited studies explored such nuanced vascular modeling. He et al. (2023) [44] and Liu et al. (2023) [5] considered vessel density as an optimization factor, but within a hard-constraint framework for vessel avoidance. Our simplified modeling of the vasculature seeks balance, ensuring the safety of the vasculature without sacrificing the efficiency of computation and clinical workflow integration, potentially offering a more clinically adaptable approach than rigid vessel exclusion while still prioritizing patient safety.

#### 4.2.4. Multi-Objective Optimization

Many existing path planning methods, as Monfaredi et al. (2024) [43] highlighted, incorporate multi-objective optimization to balance competing clinical goals. Zhang et al. (2024) [25] used fuzzy logic and Pareto fronts. Dong et al. (2022) [26] and He et al. (2023) [44] utilized Pareto optimization and NSGA-III and multi-dimensional spatial distance Pareto optimization, respectively. Liu et al. (2023) [5] employed Loose Pareto and Heptagonal Optimization. Yang et al. (2024) [17] used Pareto fronts within a constrained optimization framework. Xie et al. (2023) [45], while not explicitly using Pareto optimization, aimed to optimize multiple factors like shortest distance and vessel avoidance. Monfaredi et al. (2024) [43] reviewed various optimization techniques, including genetic algorithms and particle swarm optimization, used in path planning to address multi-objective problems. Wang et al. (2024) [21] also employed Pareto optimization in their optimal path calculation step, integrating it with the weighted summation of soft constraints. In contrast, Li et al. (2023) [15] employed a heuristic approach based on clinical planning logic rather than traditional multi-objective optimization algorithms. Our method, while implicitly optimizing for multiple factors through optical simulation (safety, shortest path, etc.), does not explicitly employ Pareto-based or other multi-objective optimization algorithms. However, the inherent nature of our optical model, encoding multiple constraints within the light propagation simulation, allows for efficient consideration of various objectives simultaneously, without the computational overhead of iterative Pareto optimization. While Pareto optimization offers explicit analysis of trade-offs and a set of Pareto-optimal solutions, our method prioritizes computational efficiency and speed by implicitly addressing multiple objectives within a single forward simulation. Further research could explore integrating Pareto optimization with our optical model to potentially offer both speed and explicit multi-objective control. This implicit multi-objective optimization through optical simulation is a key differentiator, offering a computationally efficient alternative to the explicit Pareto-based methods commonly used in path planning.

### 4.3. Limitations

The performance of our method in lung nodule segmentation was notably weaker compared to other organs, particularly evident in the higher HD95 and ASD metrics. This can be attributed to several key factors:

(1) Small Nodule Size: Lung nodules were considerably smaller in volume compared with other organs, rendering them highly susceptible to segmentation errors. Even a deviation of 1–2 voxels could lead to a substantial increase in both the ASD and HD95 metrics [46].
(2) Boundary Ambiguity and Irregular Shapes: Lung nodules often present ambiguous boundaries and irregular shapes, posing a significant challenge for accurate segmentation. This was particularly pronounced for attached nodules and ground-glass nodules, where boundaries could be connected to surrounding structures such as blood vessels or lung lobes, thereby increasing segmentation errors [47,48].
(3) Annotation Format of the LUNA16 Dataset: The annotation format inherent to the LUNA16 dataset introduced limitations in voxel-level accuracy. This dataset, used for training and evaluation, provides annotations in CSV format, defining nodules as spheres using center coordinates and diameters rather than precise voxel-level delineations [39]. This spherical CSV annotation can introduce false positive voxels when compared with a truly precise voxel-level ground truth.

These factors collectively contributed to the observed limitations in lung nodule segmentation performance, resulting in the significantly higher ASD and HD95 values compared with the segmentation of other organs in this study.

### 4.4. Future Directions

Future research will prioritize the development of improved segmentation models for both target organs and lung nodules. Enhancing the accuracy and robustness of these foundational segmentation steps is crucial for providing more precise input to our path planning algorithm, thereby potentially leading to more reliable and safer needle trajectories. This includes exploring advanced deep learning architectures and leveraging larger, more diverse datasets for training and validation of the segmentation models.

A key focus will also be on the real-world application and rigorous validation of our proposed algorithm. This involves translating the method into practical clinical settings through the development of a user-friendly interface and seamless integration with existing medical imaging systems and clinical workflows. Future studies will include comprehensive validation on diverse patient datasets and ultimately in vivo studies to evaluate the clinical performance, safety, and efficiency of our approach across a range of interventional procedures.

## 5. Conclusions

This study introduces a novel biopsy path recommendation algorithm that significantly enhances computational efficiency, segmentation accuracy, and clinical relevance. The method achieves an average runtime of 1.905 s per sample for path planning—substantially faster than the 5 s to several minutes reported in other studies—and operates without the need for specialized infrastructure. The segmentation step employs a SegResNet model with an average inference time of 63.601 s and GPU memory usage of 4321 MB; the robustness of the model is demonstrated across diverse datasets with DSC scores of 0.9122 on LUNA16, 0.8749 on Tianchi, 0.8710 on LNDb, and 0.8563 on clinical data (our internal private validation set). Moreover, the method uses optical absorption techniques to model complex structures, effectively reducing the computational burden while ensuring the avoidance of critical anatomy. Clinical evaluation further supports the utility of the algorithm: 78% of the recommended paths were rated as fully satisfactory and 22% as moderately satisfactory (with 0% unsatisfactory), and a random forest model for identifying surgeon-preferred regions achieved an AUC-ROC of 0.948, with a precision of 0.706 and a recall of 0.766. These quantifiable metrics collectively indicate that the approach successfully balances speed, interpretability, robustness, and precision, promising enhanced safety and efficiency in biopsy and ablation procedures.

## 6. Patents

The technology described in this manuscript is currently under application for a Chinese invention patent, with the application number 202411702026.0.

## Figures and Tables

**Table 1:** Quantitative evaluation results based on SegResNet.

Target	Sens	Spec	DSC	RVE	HD95	ASD
Airways	0.9268 ± 0.0818	0.9999 ± 0.0000	0.9171 ± 0.0574	0.0707 ± 0.0678	1.0085 ± 0.0252	0.2884 ± 0.0899
Body	0.9683 ± 0.0056	0.9701 ± 0.0233	0.9418 ± 0.0127	0.0564 ± 0.0299	1.3660 ± 0.3660	0.8716 ± 0.0287
Bones	0.9139 ± 0.0124	0.9981 ± 0.0003	0.9144 ± 0.0118	0.0108 ± 0.0097	1.0009 ± 0.1153	0.4371 ± 0.0067
Heart	0.9694 ± 0.0081	0.9995 ± 0.0002	0.9684 ± 0.0098	0.0089 ± 0.0089	1.4142 ± 0.7915	0.5184 ± 0.1742
Kidneys	0.9444 ± 0.0387	0.9998 ± 0.0001	0.9419 ± 0.0488	0.0379 ± 0.0579	1.0170 ± 0.4502	0.3026 ± 0.1866
Left Lower Lobe	0.9663 ± 0.0305	0.9991 ± 0.0006	0.9660 ± 0.0227	0.0186 ± 0.0325	1.0001 ± 0.2558	0.2928 ± 0.1801
Left Upper Lobe	0.9673 ± 0.0329	0.9991 ± 0.0004	0.9697 ± 0.0210	0.0151 ± 0.0287	1.0000 ± 0.4294	0.2918 ± 0.0008
Liver	0.9840 ± 0.0045	0.9993 ± 0.0005	0.9830 ± 0.0077	0.0082 ± 0.0166	1.4142 ± 0.5166	0.4298 ± 0.0728
Lung Nodules	0.5830 ± 0.3157	0.9999 ± 0.0000	0.5328 ± 0.2596	0.7777 ± 1.0616	201.9190 ± 52.7719	7.18075 ± 1.4455
Pancreas	0.8481 ± 0.1448	0.9998 ± 0.0001	0.8512 ± 0.1328	0.1084 ± 0.1394	1.7320 ± 0.3647	0.3906 ± 0.0016
Pulmonary Arteries	0.8806 ± 0.0366	0.9998 ± 0.0000	0.8826 ± 0.0275	0.0378 ± 0.0312	1.0014 ± 0.4073	0.2080 ± 0.0870
Pulmonary Veins	0.8433 ± 0.0430	0.9994 ± 0.0001	0.8576 ± 0.0264	0.0715 ± 0.0568	1.0920 ± 0.6981	0.3399 ± 0.2280
Right Lower Lobe	0.9586 ± 0.0611	0.9990 ± 0.0007	0.9557 ± 0.0847	0.0347 ± 0.1325	1.0000 ± 0.1270	0.2962 ± 0.0124
Right Middle Lobe	0.9368 ± 0.0363	0.9990 ± 0.0007	0.9193 ± 0.0479	0.0774 ± 0.1411	8.9746 ± 3.9526	1.0472 ± 0.2161
Right Upper Lobe	0.9577 ± 0.0479	0.9988 ± 0.0007	0.9554 ± 0.0309	0.0350 ± 0.0526	2.0182 ± 0.6456	0.4376 ± 0.1180
Skin	0.6685 ± 0.0177	0.9863 ± 0.005	0.6720 ± 0.2131	0.0211 ± 0.0009	28.5059 ± 10.9514	3.8390 ± 1.2075
Spine	0.9442 ± 0.0072	0.9993 ± 0.0001	0.9432 ± 0.0048	0.0086 ± 0.0058	1.0000 ± 0.4110	0.3772 ± 0.0483
Spleen	0.9674 ± 0.0409	0.9998 ± 0.0000	0.9691 ± 0.0254	0.0158 ± 0.0426	1.0846 ± 0.2497	0.3664 ± 0.0105
Vessels	0.9281 ± 0.0202	0.9993 ± 0.0001	0.9302 ± 0.0174	0.0179 ± 0.0181	1.7320 ± 0.9526	0.5909 ± 0.2047

**Table 2:** Quantitative evaluation results based on 3D-UNet.

Target	Sens	Spec	DSC	RVE	HD95	ASD
Airways	0.9321 ± 0.0885	0.9999 ± 0.0000	0.9205 ± 0.0587	0.0740 ± 0.0742	1.0000 ± 0.5919	0.3676 ± 0.1954
Body	0.9626 ± 0.0211	0.9713 ± 0.0080	0.9404 ± 0.0073	0.0471 ± 0.1248	1.3660 ± 0.3660	0.8838 ± 0.0239
Bones	0.9091 ± 0.0115	0.9983 ± 0.0003	0.9149 ± 0.0102	0.0149 ± 0.0104	1.0000 ± 0.0184	0.4314 ± 0.0065
Heart	0.9700 ± 0.0085	0.9995 ± 0.003	0.9680 ± 0.0116	0.0119 ± 0.0149	1.4142 ± 0.6882	0.5144 ± 0.0260
Kidneys	0.9515 ± 0.0314	0.9999 ± 0.0001	0.9391 ± 0.0547	0.0514 ± 0.1028	1.0000 ± 0.4149	0.3230 ± 0.0250
Left Lower Lobe	0.9690 ± 0.0201	0.9991 ± 0.0007	0.9666 ± 0.0210	0.0181 ± 0.0265	1.0208 ± 0.1559	0.3120 ± 0.1938
Left Upper Lobe	0.9688 ± 0.0317	0.9991 ± 0.0006	0.9699 ± 0.0209	0.0169 ± 0.0294	1.4142 ± 0.6981	0.3045 ± 0.0130
Liver	0.9847 ± 0.0041	0.9993 ± 0.0007	0.9826 ± 0.0085	0.0089 ± 0.0166	1.4142 ± 0.5974	0.4108 ± 0.0514
Lung Nodules	0.6775 ± 0.2702	0.9999 ± 0.0000	0.5756 ± 0.2477	1.3663 ± 4.0629	356.7180 ± 49.8263	103.8800 ± 48.1003
Pancreas	0.8673 ± 0.0981	0.9998 ± 0.0001	0.8544 ± 0.1025	0.0962 ± 0.1143	1.0085 ± 0.3120	0.3282 ± 0.0015
Pulmonary Arteries	0.8869 ± 0.0334	0.9999 ± 0.0000	0.8893 ± 0.0246	0.0420 ± 0.0323	1.0096 ± 0.5099	0.2396 ± 0.0667
Pulmonary Veins	0.8513 ± 0.0545	0.9995 ± 0.0002	0.8597 ± 0.0306	0.1005 ± 0.0708	1.0726 ± 0.3692	0.3868 ± 0.0216
Right Lower Lobe	0.9584 ± 0.0571	0.9991 ± 0.0009	0.9564 ± 0.0855	0.0395 ± 0.1658	21.5045 ± 7.8824	6.1628 ± 0.1149
Right Middle Lobe	0.9318 ± 0.0366	0.9992 ± 0.0005	0.9230 ± 0.0366	0.0641 ± 0.0737	10.1462 ± 3.7860	1.1372 ± 0.0052
Right Upper Lobe	0.9576 ± 0.0549	0.9989 ± 0.0007	0.9553 ± 0.0349	0.0365 ± 0.0602	2.0816 ± 0.9378	0.4522 ± 0.0091
Skin	0.6963 ± 0.0035	0.98534 ± 0.0000	0.6707 ± 0.2126	0.1546 ± 0.0019	33.0670 ± 15.8281	3.3824 ± 0.4396
Spine	0.9429 ± 0.0072	0.9994 ± 0.0001	0.9444 ± 0.0047	0.0076 ± 0.0064	1.0089 ± 0.1060	0.3795 ± 0.0450
Spleen	0.9742 ± 0.0133	0.9999 ± 0.0001	0.9723 ± 0.0111	0.0152 ± 0.0224	1.0086 ± 0.4226	0.3531 ± 0.0415
Vessels	0.9378 ± 0.0139	0.9993 ± 0.0003	0.9306 ± 0.0182	0.0249 ± 0.0265	1.7320 ± 0.6671	0.5475 ± 0.1030

**Table 3:** Quantitative evaluation results based on UNETR.

Target	Sens	Spec	DSC	RVE	HD95	ASD
Airways	0.9075 ± 0.0999	0.9998 ± 0.0000	0.8856 ± 0.0669	0.1018 ± 0.0994	1.0003 ± 0.4887	0.3280 ± 0.0319
Body	0.9620 ± 0.0087	0.9635 ± 0.0009	0.9296 ± 0.0310	0.0695 ± 0.0135	1.5770 ± 0.4230	1.0645 ± 0.1788
Bones	0.8908 ± 0.0203	0.9975 ± 0.0005	0.8885 ± 0.0183	0.0246 ± 0.0264	1.4142 ± 0.6456	0.5371 ± 0.0089
Heart	0.9509 ± 0.0249	0.9992 ± 0.0006	0.9475 ± 0.0249	0.0265 ± 0.0421	2.4142 ± 0.1949	0.7250 ± 0.0329
Kidneys	0.8595 ± 0.1603	0.9998 ± 0.0002	0.8760 ± 0.1331	0.1268 ± 0.1659	7.8783 ± 2.1186	1.0679 ± 0.3029
Left Lower Lobe	0.9568 ± 0.0340	0.9986 ± 0.0008	0.9525 ± 0.0325	0.0261 ± 0.0282	1.4142 ± 0.0524	0.3967 ± 0.0539
Left Upper Lobe	0.9501 ± 0.0389	0.9989 ± 0.0007	0.9571 ± 0.0257	0.0286 ± 0.0377	1.4142 ± 0.6274	0.4009 ± 0.0235
Liver	0.9753 ± 0.0112	0.9990 ± 0.0005	0.9740 ± 0.0084	0.0140 ± 0.0150	2.4142 ± 0.4351	0.6933 ± 0.1203
Lung Nodules	0.3910 ± 0.3582	0.9999 ± 0.0000	0.3675 ± 0.3239	1.8281 ± 4.5187	334.1962 ± 48.8511	329.6033 ± 18.2415
Pancreas	0.6357 ± 0.2247	0.9996 ± 0.0003	0.6391 ± 0.2082	0.4054 ± 0.8568	5.0096 ± 1.0732	0.7854 ± 0.0575
Pulmonary Arteries	0.6651 ± 0.0517	0.9995 ± 0.0001	0.6460 ± 0.0376	0.1043 ± 0.0945	14.2426 ± 5.9513	2.4166 ± 0.2596
Pulmonary Veins	0.7690 ± 0.0426	0.9989 ± 0.0003	0.7635 ± 0.0397	0.1115 ± 0.0926	2.7320 ± 0.7813	0.4889 ± 0.2309
Right Lower Lobe	0.9438 ± 0.0689	0.9985 ± 0.0010	0.9403 ± 0.0954	0.0430 ± 0.1838	1.7320 ± 0.4073	0.3946 ± 0.2175
Right Middle Lobe	0.8891 ± 0.0581	0.9988 ± 0.0005	0.8840 ± 0.0559	0.0715 ± 0.0744	7.8783 ± 1.8511	1.0683 ± 0.5540
Right Upper Lobe	0.9417 ± 0.0519	0.9983 ± 0.0010	0.9382 ± 0.0376	0.0428 ± 0.0569	2.7320 ± 0.7296	0.5497 ± 0.0423
Skin	0.6832 ± 0.0046	0.9870 ± 0.0020	0.6840 ± 0.1409	0.0043 ± 0.0015	45.3406 ± 0.2558	5.0630 ± 2.1102
Spine	0.9324 ± 0.0124	0.9991 ± 0.0002	0.9254 ± 0.0100	0.0199 ± 0.0150	1.4142 ± 0.9324	0.5290 ± 0.0732
Spleen	0.9444 ± 0.0525	0.9997 ± 0.0001	0.9486 ± 0.0393	0.0300 ± 0.0432	1.0010 ± 0.5243	0.5558 ± 0.0145
Vessels	0.8753 ± 0.0372	0.9989 ± 0.0004	0.8806 ± 0.0333	0.0413 ± 0.0325	7.9282 ± 3.141	1.4424 ± 0.2505

**Table 4:** Ablation results based on segmentation performance and efficiency.

Model Variant	Average DSC	Infer Times (s)	GPU Mem (MB)
U-Net + Base Conv (3D U-Net)	0.8921 ± 0.0045	60.288 ± 14.656	3960 ± 12
U-Net + Res Block (SegResNet)	0.9122 ± 0.0096	63.601 ± 19.671	4321 ± 16
U-Net + Transformer Block (UNETR)	0.8609 ± 0.0029	80.214 ± 40.882	6410 ± 51

**Table 5:** Quantitative evaluation results based on SegResNet on various datasets.

Dataset	Average Sens	Average Spec	Average DSC	Average RVE	Average HD95	Average ASD
LUNA16	0.9031	0.9983	0.9122	0.0668	12.7881	0.7806
Tianchi	0.9018	0.9968	0.8749	0.1616	8.5356	1.7746
LNDb	0.8952	0.9965	0.8710	0.1921	7.7988	2.1419
Clinical	0.8905	0.9969	0.8563	0.1985	8.1350	1.7342

**Table 6:** Accuracy in predicting surgeon-preferred regions.

Model	AUC-ROC	Precision	Recall	F1	Specificity	TP	TN	FP	FN
RandomForest	0.948	0.706	0.766	0.735	0.956	36	324	15	11
XGBoost	0.942	0.717	0.702	0.710	0.962	33	326	13	14
LightGBM	0.944	0.717	0.702	0.710	0.962	33	326	13	14
CatBoost	0.951	0.643	0.766	0.699	0.941	36	319	20	11
MLP_Simple	0.950	0.694	0.723	0.708	0.956	34	324	15	13
MLP_Deep	0.957	0.694	0.723	0.708	0.956	34	324	15	13
MLP_Wide	0.950	0.708	0.723	0.716	0.959	34	325	14	13
MLP_Complex	0.949	0.654	0.723	0.687	0.947	34	321	18	13
Manual	/	0.660	0.500	0.569	0.949	33	319	17	33

**Table 7:** Quantitative metrics of path parameters and computational efficiency for path planning methods.

Method	Average Depth (mm)	Average Angle (°)	Average Distance (mm)	Average Time Cost (s)
Generation of RGB Images	/	/	/	1.758 ± 0.061
RandomForest	70.774 ± 29.320	63.353 ± 11.216	3.326 ± 3.512	0.072 ± 0.022
XGBoost	72.651 ± 29.511	62.703 ± 11.177	3.154 ± 3.547	0.081 ± 0.022
LightGBM	72.247 ± 29.530	63.191 ± 11.054	3.183 ± 3.527	0.106 ± 0.028
CatBoost	73.189 ± 30.838	63.085 ± 11.487	3.292 ± 3.552	0.082 ± 0.028
MLP_Simple	76.697 ± 31.278	60.809 ± 11.826	2.892 ± 2.861	0.055 ± 0.021
MLP_Deep	73.619 ± 30.956	62.146 ± 12.175	3.115 ± 3.375	0.053 ± 0.021
MLP_Wide	74.520 ± 32.881	62.278 ± 12.600	3.027 ± 3.377	0.054 ± 0.021
MLP_Complex	74.638 ± 30.476	61.592 ± 11.985	3.323 ± 3.350	0.054 ± 0.022
Manual	74.956 ± 29.570	62.435 ± 9.511	2.883 ± 3.133	1.968 ± 0.802
Ground Truth	71.640 ± 31.556	62.696 ± 11.794	3.142 ± 3.590	/

**Table 8:** Comparison of studies on needle path planning algorithms.

Study	Existing Methods and Limitations	Proposed Improvements	Vascular Handling	Acceleration Strategy	Multi-Objective Optimization	Time Cost	Includes Organ Segmentation?
Claire Baegert (2007) [33]	Manual constraint of optimization; artificial minima risk.	Safe entry region calculation, refined via constrained triangle elimination/subdivision.	Collision detection to avoid organs, bones, and vessels.	Coarse angle discretization + local optimization.	Weighted sum: tumor volume, organ distance, and insertion depth.	~30 s	Not specified
Qi Dong (2022) [26]	Sphere models; manual weight setting; limited flexibility.	NSGA-III with adaptive morphology.	3 mm vessel dilation; boundary adjustments.	DICOM resampling + HashMap for collision detection.	NSGA-III balances efficiency and treatment.	Not specified	Not specified
Min Luo (2022) [16]	Multi-criteria and electrode planning; high cost, few clinical factors.	Multi-stage planning with risk index and potential fields.	Risk maps to avoid critical vessels.	Not specified	Not specified	<200 ms	Liver reconstruction: 5–20 min
Ruikun Li (2023) [15]	Deep learning for insertion/ablation; high cost, partial solutions.	Heuristic RFA planning with 3D-to-2D projection.	Adjusts vessel positions via projection.	Heuristic rules within projection framework.	Not specified	<3 min	Not specified
Jing Li (2023) [22]	Single/multi-needle planning and Pareto optimization; manual threshold and limited clinical constraints.	Fixed-point, ROI-based multi-needle planning.	Strict constraints to avoid vessels.	Triangular mesh reduces search space.	NSGA-II for needle placement vs. tumor coverage.	~41.4 s	Not specified
Rong-Li Xie (2023) [43]	Lung segmentation with boundary repair; repair failures, suboptimal paths.	Adaptive thresholding, morphological repair, shortest path.	Advanced segmentation defines safe zones.	Not specified	Distance-based optimization to shorten paths.	~5 s	Not specified
Qi Liu (2023) [5]	Ablation/biopsy planning; manual tasks, ignored constraints.	Relaxed Pareto with adaptive heptagonal constraints.	Geometric constraints to avoid nerves and vessels.	Voxel downsampling (1 mm) boosts efficiency.	Relaxed Pareto enables flexible decisions.	2045.3~4518.3 s	Not specified
Zhengshuai Wang (2024) [21]	Semi-automatic planning; manual interaction, sampling issues.	Cube mapping with Pareto optimization.	Cube mapping optimizes vessel avoidance.	Parallel generation of constraint maps.	Weighted Pareto improves trajectory planning.	~35 s	Not specified
Hui Yang (2024) [17]	Rigid needle planning; suboptimal targets, few clinical factors.	Dual-sphere Pareto with importance-correlation scoring.	Limits vessel intersections (≤10).	Not specified	Dual-sphere Pareto for refined planning.	~179.11 s	Not specified
Chow Wei Too (2024) [6]	Manual nodule marking, inverse kinematics; heavy manual intervention.	CNN-based lesion detection with Bayesian planning.	Dynamic vessel avoidance via Bayesian optimization.	Bayesian optimization adjusts trajectory in real time.	Not specified	<5 min	Not specified
Ziwei Song (2024) [8]	3D visualization; semi-automatic planning struggles with complex cases.	Fully automated planning with Pareto and weighted sum.	Ensures safe paths with effective vessel avoidance.	Not specified.	Combined weighted sum and Pareto optimization.	~4 min	Not specified
Jiayu Zhang (2024) [25]	Semi-automatic, centroid targeting; manual input, ignores respiratory motion.	Multi-objective fuzzy optimization.	Fuzzy logic yields zero vessel intersections.	Not specified.	Fuzzy-based Pareto for flexible decision-making.	2669.4~2477.1 s	Not specified
Ling He (2023) [44]	Constraint-based with weighted sum and Pareto; subjective weights, manual selection.	Multi-dimensional Pareto optimization.	Distance-based vessel avoidance minimizes risk.	Not specified.	Multi-dimensional Pareto for nuanced decisions.	Not specified	Not specified
Ours	Brute-force methods are slow; downsampling lowers resolution; detailed risk assessment needed.	Optical algorithm-based environment modeling.	Optical absorption simulates vessel risk.	GPU-accelerated optical algorithms for rapid computation.	Machine learning models mimic clinicians’ preferences.	1.905 ± 0.089 s	Segmentation: 63.601 ± 19.671 s

## Data Availability

The original CT data and lung nodule masks can be accessed from the LUNA16 website (https://luna16.grand-challenge.org/ (accessed on 13 March 2025)). The demonstration code and sample data are available on our GitHub repository: https://github.com/b-niu/illumination-needle-path (accessed on 13 March 2025).

## Abbreviations

The following abbreviations are used in this manuscript:

ROI	Region of Interest
DSC	Dice Similarity Coefficient
HD	Hausdorff Distance
ASD	Average Surface Distance
